# Supplementary material for: Quick Analysis of Sedimentary Ancient DNA Using quicksand
Source: Mol Biol Evol. 2025 Nov 25;42(12):msaf305. doi: 10.1093/molbev/msaf305 (PMC12684969; doi:10.1093/molbev/msaf305)
Supplement: msaf305_Supplementary_Data [file msaf305_supplementary_data.zip › supplementary_information.pdf]

Supplementary Information

Supplementary Section 1: Published Workflows in Ancient Metagenomics and sedaDNA Research

The term *ancient metagenomics* refers to the analysis of shotgun-sequenced samples for ancient DNA (see Fellows Yates et al. 2024) to emphasize the focus on multiple (unknown) sources of the DNA in the sample and the untargeted nature of shotgun-sequencing. In this section we summarize bioinformatic workflows commonly used in the field of sedaDNA or ancient metagenomics that were considered during the development of quicksand.

In the main text we focused on the advances of sedaDNA in the field of archaeology. However, the first recovery of sedaDNA came from sediment-cores used for the *reconstruction of past ecosystems* (e.g. Willerslev et al. 2003; Hofreiter et al. 2003). Since then, shotgun sequencing of sedaDNA has been widely applied to permafrost, marine, and lake sediments to investigate past biodiversity and palaeoenvironments (e.g. Kjær et al. 2022; Wang et al. 2021; Capo et al. 2021; Parducci, Nota, and Wood 2018; Pedersen et al. 2016; Liu et al. 2024). Bioinformatic workflows for ancient metagenomic data are generally agnostic to the sample type, and have been used across a wide range of substrates. In addition to workflows developed specifically for sedaDNA analysis, we also explored methods focused on ancient microbial communities - for example, those targeting host-associated microbiomes or pathogens in archaeological materials. The computational workflows generally involve:

(i) taxonomic classification, where individual DNA sequences are assigned to one or multiple reference genomes and placed on a phylogenetic tree. (ii) taxonomic binning, where sequences from the same taxonomic level (e.g., biological family) are grouped for downstream analyses. (iii) data filtering and validation. (iv) Analysis of DNA damage patterns to confirm sequence authenticity.

For the development of quicksand, we compared bioinformatic workflows and their classifiers and evaluated strategies for the authentication of results and the removal of false positives. In particular, we screened workflows published in the following fields of ancient DNA research:

1. Sediments, environmental reconstruction
2. Sediments, archaeology
3. Other substrates, (host-associated) microbial communities

In addition, we specifically surveyed aDNA studies that used tools from the Kraken family (Kraken, Kraken2 or KrakenUniq) for taxonomic classification to better

understand how false-positive assignments can be addressed and removed during downstream analyses.

Table S1.1
Overview of workflows in sedaDNA and ancient metagenomics studies

Name / Publication	Classifier	Main Filters	Substrate / Method
HOLI (Pedersen et al. 2016)	Bowtie2 + MEGAN or ngsLCA or metaDMG	Minimum number of sequences;<br>Minimum percentage of sequences;<br>Minimum aDNA damage	SG from sediments; environmental Reconstruction
Slon et al. 2017	BLAST + MEGAN. Additional mapping with BWA	Minimum number of sequences;<br>Minimum percentage of sequences	TE from sediments, (Target: mammalian and hominin mtDNA); Archaeology
Margaryan et al. 2018	**Kraken**	Minimum Kraken confidence score;<br>Minimum number of sequences;	SG from other substrate; Host-Associated Microbial Communities
HOPS (Hübler et al. 2019)	MALT	Minimum sequence complexity;<br>Shape of edit distance;<br>Ancient damage patterns	SG and TE, all substrates; Microbial Communities
Ottoni et al. 2019	**Kraken2**. Additional mapping with BWA	Minimum percentage of sequences;<br>Species blacklist;	SG from other substrate; Host-Associated Microbial Communities
"An open sourced pipeline" (Collin et al. 2020)	BLAST + MEGAN. Additional mapping with BWA	Minimum number of sequences;<br>Minimum percentage of sequences	SG from sediments; Archaeology
Schulte et al. 2021	**Kraken2**. Additional mapping with BWA	Kraken confidence score;	TE from sediments (Target: *Larix* Chloroplast); environmental Reconstruction
Courtin et al. 2022	**Kraken2**	Kraken confidence score;<br>Minimum percentage of	SG from sediments; environmental

		sequences;<br>Assignment to “likely” family	Reconstruction
HAYSTAC (Dimopoulos et al. 2022)	Bowtie2	Minimum breadth of coverage;<br>Minimum posterior probability	SG from all substrates; Microbial Communities
aMeta (Pochon et al. 2022)	Profiling with **KrakenUniq**, then MALT	Minimum number of kmers;<br>Minimum number of sequences	SG from all substrates; Microbial Communities
euka (Vogel et al. 2023)	VG Giraffe	Spurious alignment filter;<br>Minimum coverage	SG/TE from sediments (Target: tetrapod and arthropod mtDNA); Archaeology
sediMix (Xu, Zavala, and Moorjani 2025)	**CENTRIFUGE**. Additional mapping with BWA	Required assignment to Primates;	TE from sediments, (Target: Human nuclear DNA); Archaeology

Notes: The table focuses on the classifier and filter steps of published workflows. Workflows *without* a name are only described in the respective publication and not available as an executable tool or pipeline. The “Main Filters” column shows a selection of filters applied on the classified sequences to remove false-positive assignments. See the respective publications for more details.

Alignment-free classifiers in bold
Abbreviations: SG = Shotgun Sequencing, TE = Target enrichment
References: Bowtie2 (Langmead and Salzberg 2012); ngsLCA (Wang et al. 2022); CENTRIFUGE (Kim et al. 2016); metaDMG (Michelsen et al. 2022);

To date there is no executable aDNA workflow that relies on alignment-free methods for classification. The sedimix and aMeta pipelines use alignment-free classification to filter sequences before mapping or create a list of references for a subsequent alignment-based analysis. We found several studies that describe the use of alignment-free methods (see Tab. S1.1), but no general solution for the problem of false-positive taxa was described. E.g. Courtin et al. 2022 discuss the fact that they faced problems with false positive rates and recommend the use of alignment-based classifiers; Margaryan et al. 2018 state to “be cautious” with their taxonomic assignments and that Kraken should only be used as an exploratory step. In most cases, classification results are grouped on higher taxonomic levels (i.e. eukaryotes, Bacteria) or restricted to “expected” taxa (Courtin et al. 2022; Ottoni et al. 2019; Schulte et al. 2021).
Filtering strategies we selected for quicksand are further discussed in SI 4.

Supplementary Section 2: Creating Simulated Datasets

This section describes the simulated datasets used for optimizing and benchmarking quicksand. An overview of the five datasets is provided in Table S2.1. Genomic sequences used for the simulations were downloaded from NCBI using NCBI Batch Entrez (https://www.ncbi.nlm.nih.gov/sites/batchentrez), the accession numbers are listed in Table S2.2 and Supplementary Table ST1.

Table S2.1
Overview of the five simulated datasets

Dataset Size	Mammalian mtDNA	Mammalian nuclear DNA	Environmental DNA	Total number of reads
0.1	**500**	24,500	475,000	500,000
1	**5,000**	20,000	475,000	500,000
10	**50,000**	25,000	425,000	500,000
100	**500,000**	0	0	500,000
1000	**5,000,000**	0	0	5,000,000

Notes: The columns show the number of simulated sequences in each dataset. The column "Dataset Size" refers to the number of simulated mtDNA sequences (in bold) relative to the number of mammalian mtDNA sequences in an average sedaDNA MiSeq dataset (see SI 2.1).

We simulated paired end (PE) Illumina MiSeq sequencing reads with universal Illumina sequencing adapters using gargammel `v1.1.2` with the flags `-ss MS` (Renaud et al. 2016). Gargammel is designed to simulate sequences from a single "endogenous" genome with the option to add bacterial contamination. To simulate metagenomic datasets, with sequences coming from multiple (mammalian) genomes, we treated all downloaded genomes as "bacterial contamination" by placing them as indexed fasta file in the `bact/` directory of the gargammel working directory and executing gargammel with the `--comp 1,0,0` flag.

Each of the five datasets was created three times independently, incorporating no, medium or high levels of aDNA deamination damage (C-to-T substitutions) in the terminal five base-pairs of each DNA sequence using the gargammel `-matfile` option. The matrix files for medium and high damage were the ones used for the verification of *euka* by Vogel et al. 2023 and are based on patterns observed in ~4000 years old neolithic samples (Günther et al. 2015; C-to-T substitution frequencies ranging from 1% at the 5th to 17% at the terminal base position) and ~7000 years old mesolithic samples (Olalde et al. 2014; 14-33%), respectively. The read length distribution,

provided to gargammel with the `-f` flag followed the distribution observed in shotgun sequences of a Neanderthal sample from *Vindija Cave* in Croatia (Prüfer et al. 2017) with sequence lengths restricted to a minimum of 35 and a maximum of 100 base pairs (`--minsize 35` and `--maxsize 100` flags).

The creation of the simulated MiSeq PE sequencing reads was followed by Illumina adapter-removal and overlap-merging to full-length DNA molecules using leeHom (Renaud, Stenzel, and Kelso 2014) with the `--ancientdna` flag to allow partial overlap of simulated reads. The full-length sequences of the mammalian mtDNA, mammalian nuclear DNA and environmental background DNA were simulated for each dataset and damage profile independently and combined into FASTQ files using the unix 'cat' command.

2.1 Mammalian mtDNA and Nuclear Genomes

Sequences were simulated from Mammalian families found in Pleistocene cave sediments, namely, Hominidae (Neanderthal), Elephantidae (Mammoth), Hyaenidae (Cave hyaena), Bovidae (Bos taurus) and Suidae (Sus scrofa) in proportions adopted from Slon et al. 2017 (see Table S2.2). The number of simulated mtDNA sequences increased across the five datasets from 500 to 5,000,000. The number of 5,000 mammalian mtDNA sequences was picked as baseline (Dataset Size 1) as it corresponds to the average number of mammalian mtDNA sequences in target-enriched sedaDNA datasets sequenced with ~500,000 reads (~4,800 sequences, for samples published in Slon et al. 2017).

Table S2.2
Mammalian genomes used as source for gargammel (NCBI accession numbers)

Mammalian Family	mtDNA Genome	Nuclear Genome	Proportion
Hominidae	KC879692.1	GCF_000001405.25	0.01
Elephantidae	EU153448.1	GCA_000001905.1	0.04
Suidae	KC469586.1	GCA_000003025.6	0.15
Bovidae	DQ124371.1	GCA_000003055.5	0.3
Hyaenidae	JF894377.1	GCA_008692635.1	0.5

Notes: The same proportion of sequences per genome was used in all dataset sizes.

2.2 Environmental Background DNA

We simulated ancient DNA sequences from bacteria, archaea, fungi, plants and viruses, as proposed by Vogel et al. 2023 to explore the effect of ‘background DNA’ (DNA from sources that are not represented in the constructed reference database) on the quicksand results. Datasets with less than 500,000 mammalian mtDNA sequences were filled up with simulated background DNA as shown in Table S2.3. The full list of genomes used as source for the background DNA is provided in supplementary table T1.

Table S2.3
Number of “ background DNA” sequences simulated per dataset.

Dataset Size	Bacteria	Archaea	Fungi	Viridiplantae	Viruses
0.1	400000	35000	30000	8000	2000
1	400000	35000	30000	8000	2000
10	350000	35000	30000	8000	2000

Notes: See Supplementary Table ST1 for a list of reference genomes used

Supplementary Section 3: Optimizing Database Kmer Size for sedaDNA Datasets

The optimal kmer length was determined using three criteria:

1. The *accuracy,* defined as the number of true positive sequence classifications divided by the total number of identified sequences (see main text)
2. *The sensitivity*, defined by the number of true positive sequence classifications (see main text)
3. The number of identified false positive families

3.1 Number of False-positive families

We quantified the total number of false-positive family assignments in KrakenUniq (not quicksand as a whole) for each dataset, kmer size, and damage profile. Fig. S3.1 and S3.2 show that the number of false positive family assignments is primarily driven by the DNA damage profile (panels A, B and C) and the size of the simulated dataset. The kmer size only mattered when a unique kmer filter was applied to reduce taxonomic noise (see SI 4). In that case, smaller kmer-sizes show an increased number of false positives, because more unique kmers are generated from the same sequence, making it more likely to pass the filtering thresholds.

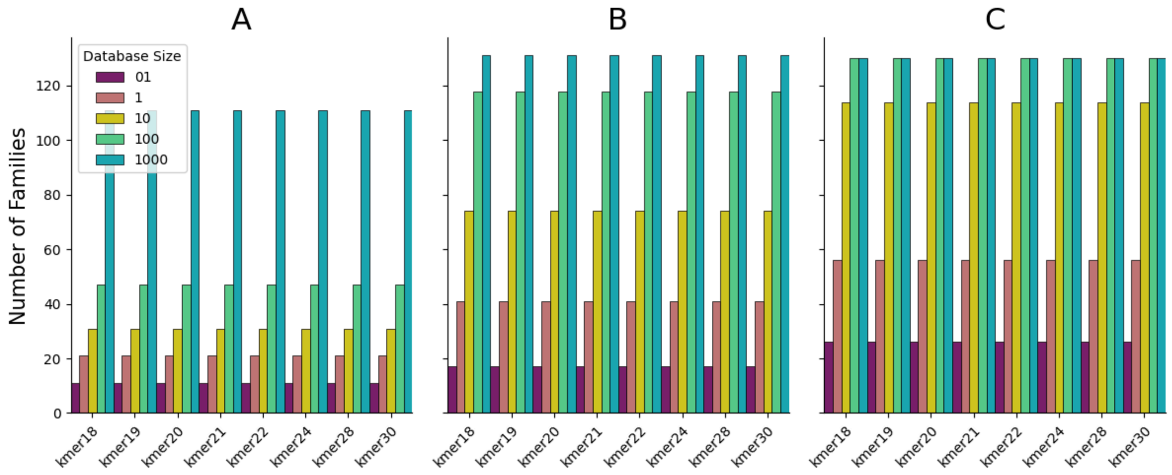

Figure S3.1 | Number of false positive **KrakenUniq** family assignments across different kmer sizes without the unique kmer-filter (see SI 4). Results for (A) undamaged, (B) medium damaged, and (C) highly damaged datasets. The barplots show the number of false-positive families found in each dataset with the respective kmer-size. Notably, 131 is the maximum number of mammalian families detectable in the database. Dataset size: see Tab. S2.1

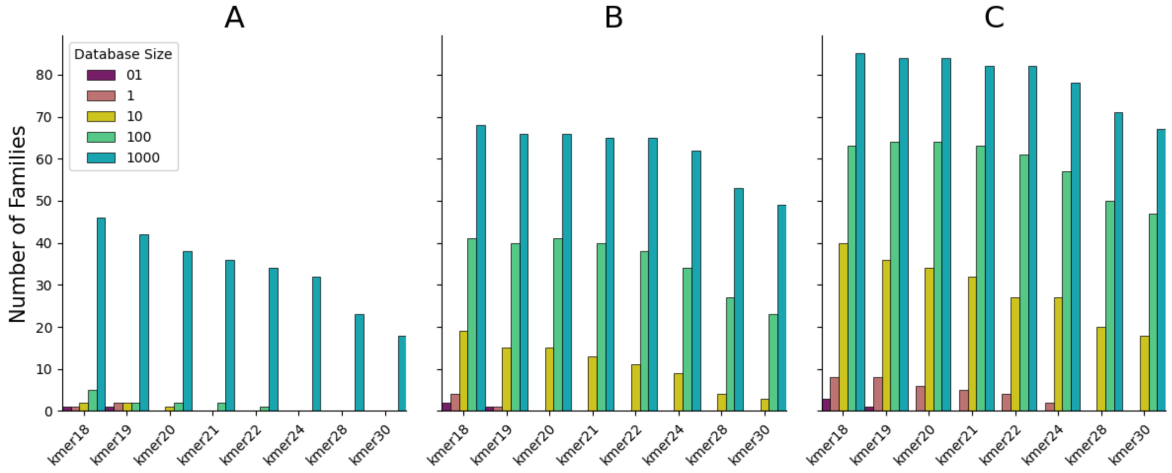

Figure S3.2 | Number of false positive **KrakenUniq** family assignments across different kmer sizes with an applied threshold of 129 unique kmers per family (see SI 4). See Fig. S3.1 for figure caption.

The high number of false-positive assignments is expected, as kmer-based methods are known to generate a substantial number of background identifications. It shows the importance of filtering KrakenUniq identifications and applying additional downstream filters to eliminate false-positive family assignments. We conclude that kmer size alone does not substantially influence the rate of false-positive assignments and that the counting of unique kmers is not sufficient as a standalone filter.

Supplementary Section 4: Implementing Filters for the Removal of False-positive Family Assignments

The identification of false positive assignments is a well known problem for metagenomic analyses in general and for kmer-based classifiers in particular. To address this, several filtering strategies have been developed (see Table S1.1 for overview), which generally fall into the following categories:

1. **Quantity-based filters**: Filters that remove classifications which do not pass a predefined threshold (e.g minimum sequence counts or percentages of counts)
2. **Coverage-based filters:** Filters that assess the evenness of coverage in the alignment. Uneven coverage indicates mismapping, e.g. sequences clustering at conserved regions in the reference genome.
3. Incorporation of **biological expectations** - i.e. removal of *unlikely* species from the assignment based on known biological constraints.

We explored both quantity-based (SI 4.2) and coverage-based (SI 4.3) filters and established a set of recommended thresholds. However, most filters are not applied automatically within the pipeline. Instead, the relevant values for filtering are reported in the final summary report and need to be applied by the user. These statistics are calculated from the BWA alignments after PCR duplicate removal. We therefore aim to pass as many KrakenUniq assignments as possible to the mapping step.

However, KrakenUniq assigns sequences to all possible families in large and highly damaged datasets (Fig. S3.1). Forwarding all these assignments to the mapping step negates the speed advantage of kmer-based classification. In order to keep runtime low and to reduce the generation of non-informative (“junk”) output files, we, by default, apply a low unique kmer filter of 129 kmers per family to remove false-positives prior to mapping (SI 4.1).

4.1 Removal of KrakenUniq background identifications using unique kmers

In contrast to Kraken or Kraken2, KrakenUniq reports the number of unique kmers found for each taxonomic classification, a value that serves as a proxy for the breadth of coverage in a hypothetical alignment with the reference genome in the database. Filtering assignments by the number of unique kmers was proposed by the authors of KrakenUniq, who recommended a threshold of 1,000 kmers per species to eliminate “background identifications”, a threshold confirmed by the authors of aMeta (Pochon et al. 2022; and recently Oskolkov 2025). We believe that this threshold is too strict for quicksand - given that additional filtering steps are applied downstream, following actual sequence alignments.

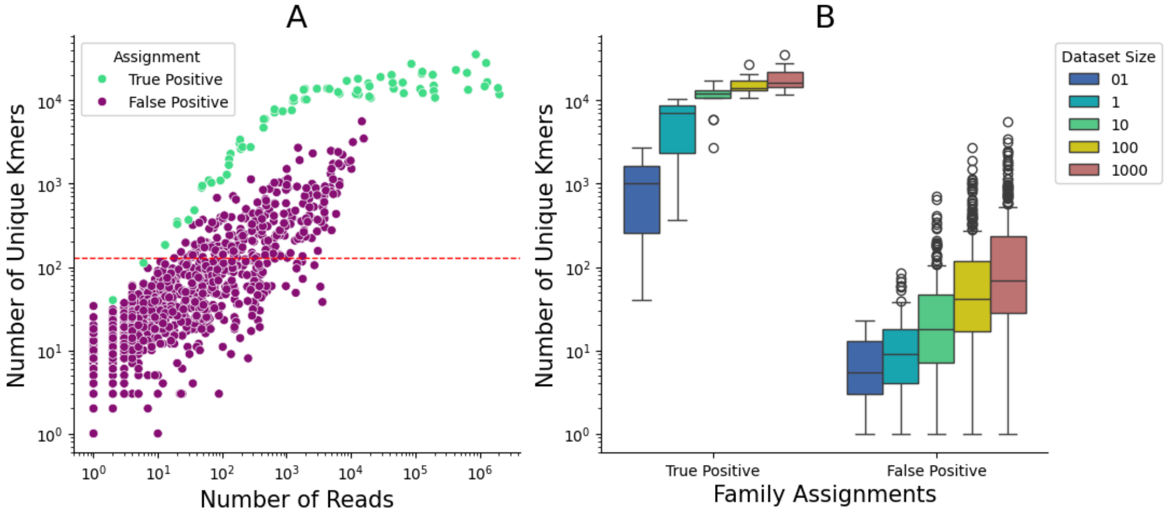

Figure S4.1 | Number of unique kmers assigned for each family in the simulated datasets (kmer-size 22). (A) By the number of reads, the red line indicates the default cutoff of 129 kmers. (B) By true and false positive family assignment, stratified by the dataset size (see Tab. S2.1). Boxplot formatting follows Fig.2;

In the simulated datasets, we observed that the number of unique kmers assigned to false-positive families increased with dataset size, rising from a median of 5 (max: 23) in the smallest to 69 (max: 5,616) in the largest dataset (Fig. S4.1B). This suggests that a percentage-based filtering threshold, e.g. one that removes families with fewer than 1% of unique kmers, is more effective as it does not depend on the size of the dataset. However, it should be noted that a percentage-based threshold may lead to the loss of true positive families especially in cases where the distribution of sequences across different families is highly uneven.

Families filtered from the KrakenUniq assignment *are not listed* in the summary report of quicksand (only in the KrakenUniq reports generated for each dataset). Therefore, to avoid the "silent" removal of potential true-positives, we implemented a minimally invasive static cutoff of 129 unique kmers, corresponding to ~150bp of covered sequences in the reference genome at a kmer-size of 22. This threshold successfully removed 100% of the false-positive families in the two smallest datasets and 75% and >50% in the second-largest and largest datasets, respectively. However, in the smallest dataset this filter also removed the Hominidae family (with five sequences), setting quicksand's default detection threshold to approximately ten sequences per biological family. While this threshold is fully customizable via the --krakenuniq_min_kmers flag, all subsequent filtering steps were tested with a unique kmer filter of 129 already applied. We discuss the use of lower thresholds to increase sensitivity and to enable the screening of shallow shotgun libraries in SI 6 and SI 8.2.3, respectively

4.2 Minimum Percentage of unique Sequences per Family (PSF) Filter

Filters based on a minimum percentage of mapped and deduplicated sequences assigned per biological family ("PSF") have shown to be effective in removing misidentified sequences (Tab S1.1). To explore the impact of this approach on the quicksand identifications, we compared the relative contribution of correctly and incorrectly identified families in the simulated data to the total number of mapped and deduplicated sequences (Fig. S4.2).

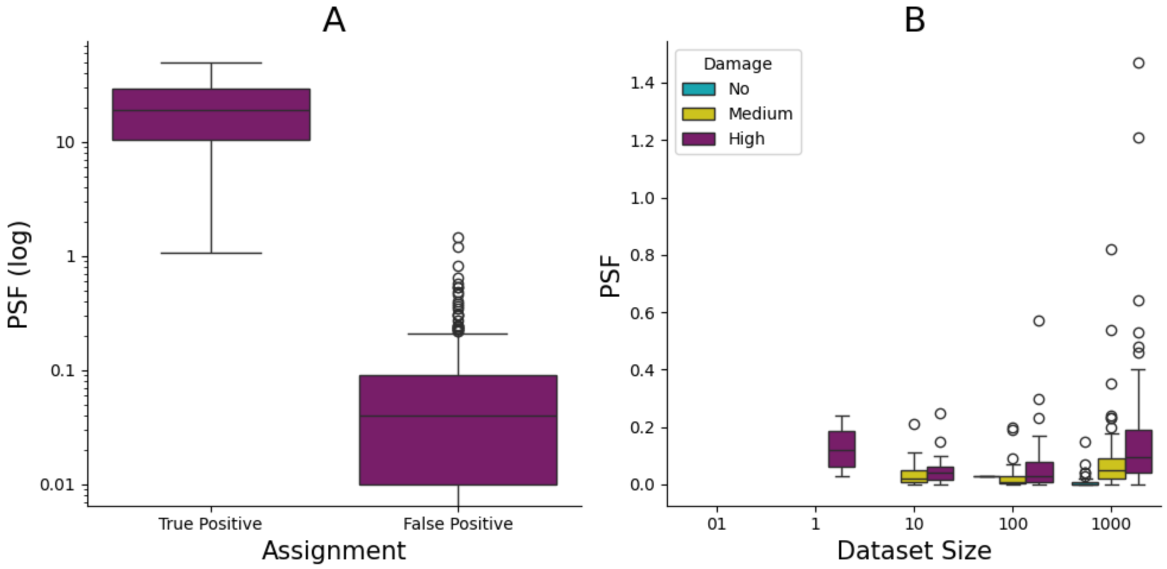

Figure S4.2 | Percentage of mapped and deduplicated sequences assigned to correctly and incorrectly identified taxa. **(A)** Distribution of PSF-values in true positive and false positive assignments (log-scaled). (**B**) Distribution of PSF-values in false positive taxa only, stratified by dataset size and damage profile. For dataset sizes, see Tab S2.1. Boxplot formatting follows Fig.2.

False-positive families are supported by a low PSF (median below 0.1%), but are generally more abundant in the larger and more damaged datasets. The PSF values are listed as 'FamPercentage' in the quicksand final summary report. We recommend a PSF threshold of at least **0.5%**.

4.3 Minimum Proportion of Expected Breadth (PEB) Filter

As a second filter, we evaluated the evenness of coverage of mapped sequences along the reference genome for each family-level assignment. High evenness of coverage indicates that sequences are randomly distributed across the reference genome, which is expected when the source of the mapped DNA is closely related to the reference, such as from the same or a closely related species. In contrast,

sequences that originate from more diverged sources may only map to conserved regions, resulting in clustered alignments and low evenness of coverage. Filtering based on evenness of coverage can therefore help identify and remove false positive taxonomic assignments.

The parameter reported in the quicksand final summary report for evaluating coverage evenness is the **proportion of expected breadth**. To understand this metric, it is necessary to define two key statistics calculated from an alignment:

- **Breadth of coverage**, defined as the proportion of the reference genome covered by at least one sequence.
- **Genomic coverage** (or depth of coverage), defined as the average number of times each base in the reference genome is covered by mapped sequences.

Under the assumption of random mapping to the correct reference genome, the breadth of coverage is a function of the genomic coverage and can be calculated using the formula empirically determined by Olm et al. 2021 (1).

$$(1)\ breadth\ of\ coverage\ =\ 1\ -\ e^{-0.883\ *\ coverage}$$

We refer to the calculated breadth of coverage as the **expected breadth of coverage**, as it assumes mapping to the correct reference genome. To evaluate deviations from this expectation, we calculated for each family the **proportion of expected breadth (PEB)**, defined as the ratio of the observed to the expected breadth of coverage. We found that the PEB was a suitable metric for distinguishing true from false positive family assignments (Fig. S4.3). For correct family assignments, the observed breadth of coverage matches the expectations (PEB around 1), while the false-positive families show PEB values between 1 and 0.2. We found that false-positives with low genomic coverage (<0.1x), match the expected breadth more often, presumably due to the small number of mapped sequences appearing randomly distributed, whereas only higher sequence counts reveal the clustering of sequences.

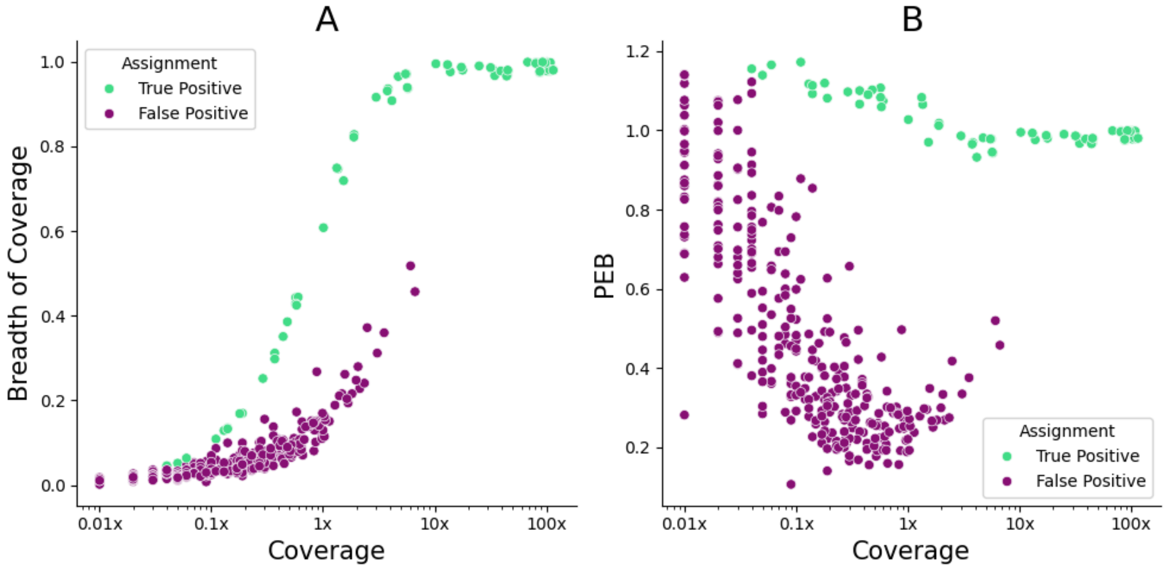

Figure S4.3 | True and False positive family assignments stratified by the genomic coverage of each alignment and (A) the breadth of coverage, (B) the proportion of expected breadth (PEB)

Based on the simulated datasets, we recommend removing family-level assignments with a proportion of expected breadth (PEB) below 0.5. Encouragingly, when applying the recommended filter cutoffs for both PSF and PEB, all false positive taxa were successfully removed from the quicksand results.

Supplementary Section 5: Benchmarking quicksand against BLAST/MEGAN, HOLI and *euka*

This section describes how the BLAST/MEGAN, the HOLI and the *euka*-based pipelines were executed for the benchmarking of quicksand and how the respective databases were constructed.

5.1. Creation of Databases

To ensure that the output of the different benchmarking-pipelines are comparable, we used the same set of mtDNA reference genomes for the construction of the required databases for BLAST/MEGAN, quicksand and HOLI.
The reference database used with the BLAST/MEGAN pipeline described by Slon et al. 2017 was not possible for us to update without substantially updating and altering the pipeline workflow. We therefore restricted the quicksand and HOLI databases to the 796 mammalian mtDNA genomes contained in the already existing BLAST/MEGAN database.

BLAST/MEGAN reference database

For BLAST we used the same reference-database described in Slon et al. 2017, consisting of 796 mammalian mtDNA genomes that were downloaded from NCBI RefSeq (O'Leary et al. 2015) in January 2016. The ABIN file required for the MEGAN step (`nucl_acc2tax-Nov2016.abin`) was downloaded from the MEGAN6 website in 2016. We provide both files on the MPI EVA FTP-server:

```
wget http://ftp.eva.mpg.de/quicksand/benchmark/RefSeq_MammalsMT.fasta
wget http://ftp.eva.mpg.de/quicksand/benchmark/nucl_acc2tax-Nov2016.abin
```

quicksand reference database

We created a fresh quicksand-database restricted to the genomes in the BLAST/MEGAN database by using the `--include` flag with the list of NCBI accession IDs in the BLAST/MEGAN FASTA-file.

```
nextflow run mpieva/quicksand-build \
      -r v3.2 \
      --include $(grep ">" RefSeq_MammalsMT.fasta | cut -d '|' -f 4
|
      sort | uniq | paste -sd,) \
      --outdir refseq \
      -profile singularity
```

HOLI reference database

The HOLI pipeline requires a bowtie2-index for classification, as well as NCBI taxonomy files for the metaDMG-step. We constructed a bowtie2-index from the mtDNA genomes used for the quicksand and BLAST/MEGAN pipelines

```
mkdir bowtie2-index && cd bowtie2-index

cat ../refseq/genomes/*/*.fasta > mammaliaMT.fasta

singularity exec
https://depot.galaxyproject.org/singularity/bowtie2:2.5.4--he96a11
b_5  \
     bowtie2-build \
     --threads 50 \
     mammaliaMT.fasta mammaliaMT
```

The metaDMG step requires as input the NCBI taxonomy files (names.dmp, nodes.dmp and accession2taxid). The .dmp files were already downloaded by the quicksand-build pipeline; we therefore only downloaded the accession2taxid file, as specified in the metaDMG documentation.

```
wget
https://ftp.ncbi.nlm.nih.gov/pub/taxonomy/accession2taxid/nucl_gb.
accession2taxid.gz;

gunzip nucl_gb.accession2taxid.gz;
```

euka reference database

The *euka* database cannot be customized and was downloaded as indicated in https://github.com/grenaud/vgan/wiki/euka#quick-start.

```
wget -nc -l1 --recursive --no-parent -P euka_dir/ \
     ftp://ftp.healthtech.dtu.dk:/public/euka_files/
```

5.2 Running the BLAST/MEGAN-based Pipeline

For benchmarking quicksand, we implemented a Nextflow-based version of the BLAST/MEGAN workflow outlined in Slon et al. 2017 (https://github.com/merszym/blastmegan_nf).

In brief, DNA sequences are assigned to a mammalian family using BLAST and the last-common-ancestor algorithm implemented in MEGAN. For each detected family, sequences are re-mapped with BWA to all available mtDNA reference genomes from that family. The alignment with the highest number of mapped sequences is then selected for downstream processing. Alignments are filtered (removing unmapped reads and those with mapping quality below 25) and PCR duplicates are collapsed into unique DNA molecules using bam-rmdup. Deamination frequencies are calculated

from the resulting alignments. The re-implemented pipeline allows the processing of multiple input-BAM files in parallel and to generate a summary report matching the one created by quicksand.

For the mapping with BWA, we used the same set of mtDNA reference genomes used for classification. We executed the nextflow pipeline with the following command:

```
nextflow run merszym/blastmegan_nf \
      --split INPUT \
      --acc2taxid nucl_acc2tax-Nov2016.abin \
      --database RefSeq_MammalsMT.fasta \
      --genomes refseq/genomes/ \
      --pseudouniq_filterflag 1 \
      --pseudouniq_mindup 1 \
      -profile singularity
```

We ran the BLAST/MEGAN pipeline with the flags `--pseudouniq_filterflag 1` (remove only unmerged reads, keep unmapped reads) and `--pseudouniq_mindup 1` (retain sequences that appear only once).

Post-processing of the pipeline-output followed Slon et al. 2017, with a Minimum percentage abundance threshold per sample and family (PSF-filter) of 1%

The summary-reports of the BLAST/MEGAN pipeline for the simulated datasets are provided in the analysis github-repository. (https://github.com/merszym/quicksand-paper-analyses/tree/master/assets/BENCH/blastmegan_nf).

5.3 Running the *euka*-based Pipeline

Since *euka* does not support batch processing of multiple files and requires input in FASTA format, we implemented a minimal pipeline around euka in Nextflow (https://github.com/merszym/euka_nf). We added the BAM-to-FASTA conversion, enabled parallel processing of *euka* for multiple input files, and compiled a final summary table for comparison with quicksand. We ran *euka* with default parameters, including the `--outFrag` flag, to get assignment statistics for each input sequence.

For our convenience, we added the option to use the NCBI taxonomy files (names.dmp, nodes.dmp) to add taxonomic level annotations based on the euka taxName column. For that we used the files downloaded with the quicksand-build pipeline (SI 5.1).

We executed our euka-based pipeline with the following command:

```
nextflow run merszym/euka_nf \
      --split INPUT \
      --euka_dir euka_dir \
```

```
      --taxonomy refseq/kraken/Mito_db_kmer22/taxonomy \
      -profile singularity
```

No further post-processing of the pipeline output was conducted.

The summary-reports of the euka-based pipeline for the simulated datasets are provided in the analysis github-repository (https://github.com/merszym/quicksand-paper-analyses/tree/master/assets/BENCH/euka_nf).

5.4 Running the HOLI Pipeline

For a comparison of quicksand with HOLI (Pedersen et al. 2016), we implemented the 'HOLI principles' in Nextflow (https://github.com/merszym/HOLI_nf) to handle the processing of multiple BAM files in parallel and to create a quicksand-like summary report.

Unfortunately, HOLI is not a single executable tool or pipeline, but a set of 'principles' described with variations in different publications (e.g. Pedersen et al. 2016; Wang et al. 2021, Kjær et al. 2022) and across several github repositories. The core of HOLI is a competitive mapping step with bowtie2, followed by a last common ancestor (LCA) step and DNA damage pattern analysis. The tool used for the LCA step changed over time (from MEGAN to ngsLCA to metaDMG) and both sga (Simpson and Durbin 2010) and vsearch (Rognes et al. 2016) have been described for data pre-processing.

We based our implementation of HOLI on the KapCopenhagen-analysis described in Kjær et al. 2022, as the authors not only provide the HOLI bash-scripts, but also the R-scripts used for the postprocessing of the HOLI output on github (https://github.com/miwipe/KapCopenhagen). The only difference is the metaDMG step that we implemented according to https://github.com/miwipe/Holi, as we were unable to run the core version of metaDMG in a software-container and used a self-compiled and containerized metaDMG-cpp version instead.

In summary, our implementation of HOLI converts input BAM files to FASTQ, which then undergo three different preprocessing steps with sga (preprocess, index and filter). The filtered FASTQ files are then mapped with bowtie2 to the provided index, followed by sorting of the alignments with samtools and metaDMG-cpp analysis (lca, dfit and aggregate).

Following the creation of the bowtie-2 index (SI 5.1), we ran the HOLI benchmarking pipeline with the following command:

```
nextflow run merszym/HOLI_nf \
      --split INPUT \
      --database bowtie2-index
      --names refseq/kraken/Mito_db_kmer22/taxonomy/names.dmp \
      --nodes refseq/kraken/Mito_db_kmer22/taxonomy/nodes.dmp \
```

```
    --acc2tax nucl_gb.accession2taxid \
    -profile singularity
```

For the post-processing of the pipeline output we adapted the strategy from Kjær et al. 2022 (https://github.com/miwipe/KapCopenhagen/blob/main/scripts/KapKmetaDMG6AnimalFamilies.R): We first applied an initial filtering of the HOLI output to assignments with 5 or more sequences and restricted assignments to the 'family' rank. Then we applied the "half of the median" filter, keeping only families with more assigned sequences than half the median of all family sequences. (e.g. If three families have 100, 50, and 5 assigned sequences, the median is 50 and the cutoff is 25 - so the family with 5 reads is removed). In the last step, we only kept families that reached a minimum percentage abundance of 1% (PSF-filter).

Compared to the filtering described in Kjær et al. 2022, we did not apply an aDNA damage filter, as this was not part of the benchmarking and also not implemented for the quicksand- euka or BLAST/MEGAN pipelines. We also applied the comparative filters (e.g. the "half of the median" filter) only to assignments from the same sample, to avoid filtering assignments in the smaller datasets based on a median that includes the large datasets. For the same reasons we dropped the 'replication' filter (requiring a family to be seen in at least 3 samples) and the 'half of the median' filter for the total number of assigned sequences per sample (which was keeping only *samples* with a total number of sequences above half the median of assigned sequences in all samples).

The summary-reports of the HOLI pipeline for the simulated datasets are available in the analysis github-repository. (https://github.com/merszym/quicksand-paper-analyses/tree/master/assets/BENCH/HOLI_nf).

Supplementary Section 6: Detecting the Hominidae family based on low sequence counts

As outlined in SI 4.3, we implemented a filter-threshold of 129 unique kmers for KrakenUniq family assignments to remove background identifications and to reduce runtime in the downstream mapping step. This threshold removed the true positive Hominidae assignment in the smallest simulated dataset with five human mtDNA sequences. Here we explored the effect of dropping the unique kmers filter for detecting families supported by low sequence counts.

Specifically, we ran additional simulations to address two questions:

1. How does quicksand perform when analyzing datasets consisting of fewer than 30 human sequences?
2. How reliable are low-read human assignments with another dominant mammalian family present in the sample?

6.1 Analyzing Low-count Hominidae Sequences

We tested how consistently quicksand found true-positive families based on low sequence quantities. From the pool of 50,000 highly damaged simulated Neanderthal sequences (*Dataset size 1000, high damage profile; see SI 2*), we repeatedly (100 replicates each) subsampled between 5 and 30 random sequences into separate files (2600 in total) that were then analyzed using quicksand.

Without applying the unique kmers filter, quicksand was able to detect the Hominidae family in 90% of the subsamples with only five mtDNA sequences, and in 100% of subsamples with seven mtDNA sequences (Fig. S6.1C). However, not all false positive families were removed by the downstream filters. As shown before (see SI 4.3), families with low numbers of sequences escape PEB filtering, as the distribution of mapped reads looks sufficiently random to match the expectation of a true positive family. False positives (Cercopithecidae and Muridae), passing both the 0.5% PSF and the 0.5 PEB filter already appeared in replicates with as few as ten input sequences (Fig. S6.1A,B).

While only approximately 17% of the subsamples with 30 input sequences showed an ‘Ancientness’ rating of ‘+’ or ‘++’ (see main text), we observed stable mean terminal deamination rates (ranging from 25% to 35%, with 95% confidence intervals between 20% and 40%; Fig. S6.1C) across all subsamples with 16 or more sequences. These point estimates match the expected deamination rates of ~30% at both the 3′ and 5′ ends. Samples with fewer sequences exhibited greater variation in terminal deamination rates, with mean values ranging from 25% to 40% and 95% confidence intervals spanning from 15% to 50% for both 5′ and 3′ C-to-T deamination rates.

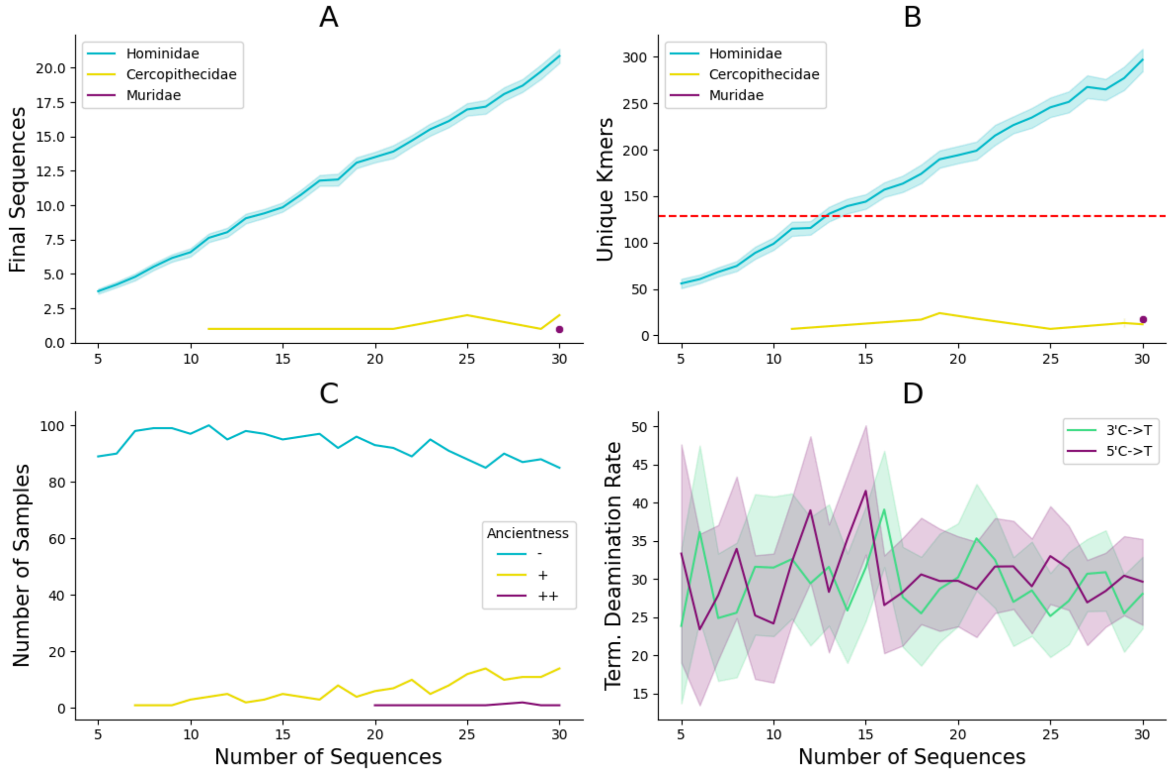

Figure S6.1 | Results of family assignments with low sequence counts. All plots are stratified by the number of Hominidae sequences put into quicksand (100 replicates each). (A) Mean number of final sequences (mapped, deduplicated, and bed-filtered) obtained for each assigned family. (B) Mean number of unique kmers obtained for each family. Red line indicates the default 129 unique kmer threshold. (C) Count of samples with the respective 'Ancientness' labels. (D) Mean 3′ and 5′ terminal C-to-T deamination rates (point estimates). Line plots in panel A,B and D show the mean (bold line) and the 95% confidence interval for repeated values (n = 100).

In summary, we show that quicksand is able to detect families even with as few as five input sequences. While the statistical power of these assignments is too low to label them as being 'Ancient' ('++' or '+'), detected deamination rates (point estimates) match the expected terminal deamination rates of the simulated data. We note that the PSF and the PEB filters alone are not sufficient to remove false-positive assignments at these low read counts. However, lowering the unique kmer threshold can be useful for screening low-yield samples that would be classified as negative under the default settings. A real-data example demonstrating this approach for screening shallow shotgun-sequencing data is provided in SI 8.2.3.

6.2 Analyzing Low-count Hominidae Sequences in the Presence of a High-count Hyaenidae Family

To test whether quicksand can detect low-count Hominidae sequences in the presence of a high-count Hyaenidae background, we added between 5 and 15

Hominidae sequences (randomly drawn as described in SI 6.1) to 2.5 million highly damaged Hyaenidae sequences previously simulated (*dataset size 1000; high damage profile;* SI 2), resulting in eleven datasets with identical Hyaenidae content but different numbers of random Hominidae sequences. We then analyzed these datasets using quicksand with default parameters, except with the unique kmer filter set to 0.

With the unique kmer filter omitted, all 131 mammalian families were identified by quicksand (Fig. S6.2A). As previously reported (SI 4), these false-positive families represented between 0.01% and 5% of the "final" (mapped, deduplicated, and bed-filtered) sequences (Fig. S6.2C). Between 42 and 60 sequences were assigned to the Hominidae family, up to six times the number of human sequences actually present in the dataset (Fig. S6.2B).

When applying the default PSF and PEB filters (Fig. S6.2C), quicksand removes the Hominidae family together with the false-positive family assignments from the summary report. Notably, the Hominidae family would have passed the threshold for 129 unique kmer (Fig. S6.2D).

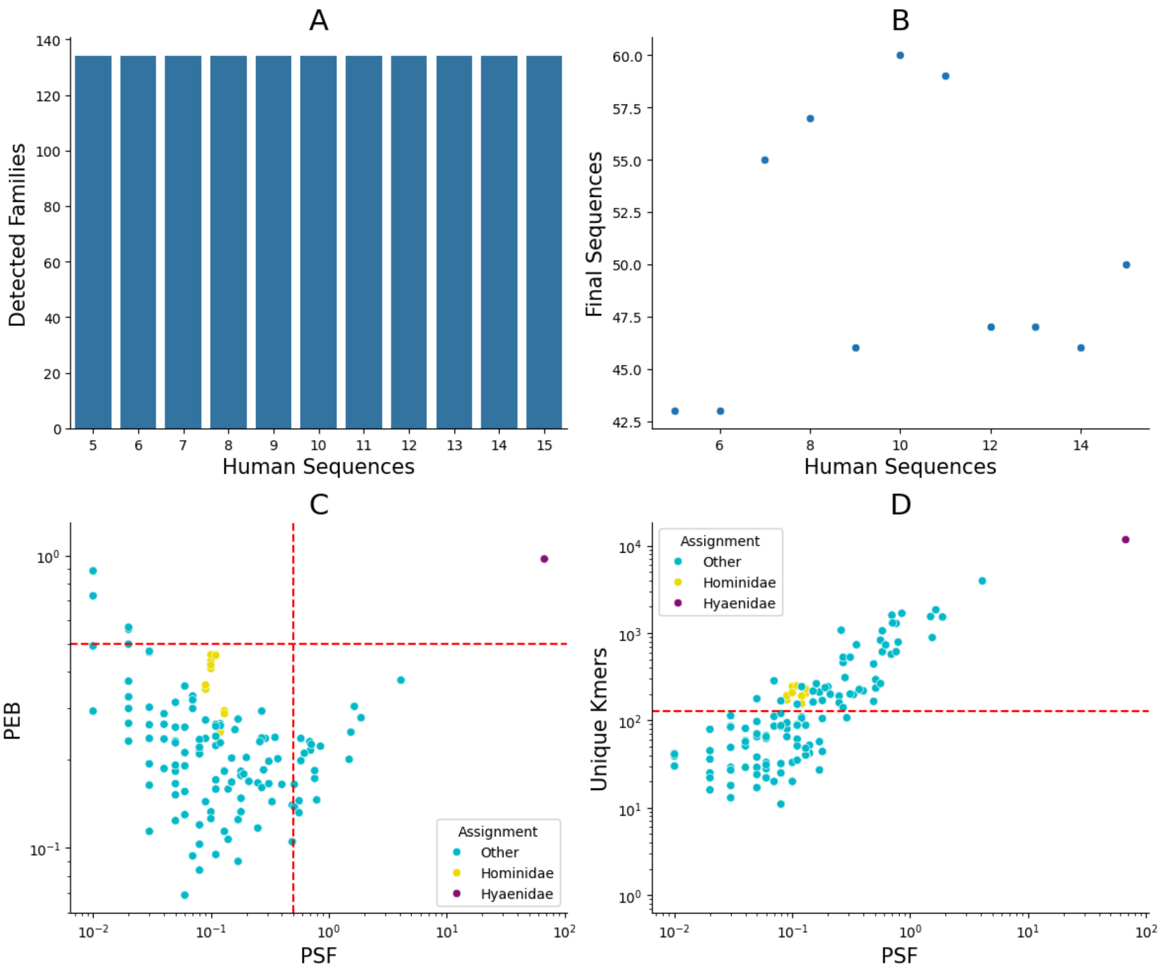

Figure S6.2 | Hominidae assignments in the context of high-abundant Hyaenidae sequences. Plots A and B are stratified by the number of Hominidae input sequences (one dataset per group), while plots C and D summarize all datasets. (A) Number of detected

families in each dataset. (B) Final sequences (mapped, deduplicated, and bed-filtered) obtained for the Hominidae family assignment in each dataset. (C) Family assignments, stratified by the percentage of unique sequences per family (PSF) and their respective PEB. Red lines show the default quicksand filter thresholds for these two values. (D) Family assignments, stratified by PSF and the respective unique kmer count. The red line shows the default 129 unique kmer threshold.

We conclude that in the presence of an over-abundant mammalian family, the spill-over of sequences into true-positive families prevents the detection of low-count true-positive families, as misclassified sequences dominate the respective assignments. As a result, such families show a lower PEB-value and are removed by the default quicksand filters.

While SI 6.1 suggests increased sensitivity when lowering the unique kmer filter, we consider a cutoff of 129 to be appropriate for the default quicksand runs, as it effectively removes a big portion of the KrakenUniq background identifications. If higher sensitivity is required, lowering the unique kmer cutoff can be useful. However, this is only recommended for datasets in which mammalian mtDNA sequences constitute only a small fraction of the total data (e.g., in shotgun-sequenced samples; see SI8.2.3). In the presence of overly high-yielding mammalian families, low-count assignments should be handled with caution, as they are potentially a mix of true and false positive sequences and require further validation downstream.

Supplementary Section 7: Analyzing sedaDNA from Denisova Cave, Main Chamber with quicksand v2.3

We verified the performance of quicksand v2.3 and the kmer-size of 22 using a dataset published in Zavala et al. 2021 (see main text). In this section we show how the filter-thresholds we established with the simulated data perform on real data.

7.1 Filtering the mammalian mtDNA capture libraries

We reanalyzed the 274 mammalian mtDNA capture libraries published as “initial screening” in Zavala et al. (2021). The mammalian capture panel used (AA75) was designed from 242 complete mammalian mtDNA genomes as a generic capture set intended to enrich samples for mammalian mtDNA fragments. This generic enrichment allows the assumption of a random distribution of mapped reads across the reference genome for each mammalian alignment, as required by the expectation-based PEB filter.

As determined for the simulated data, we applied both the 0.5% PSF filter and the PEB filter of 0.5 (Fig. S6.1). To assess the performance of these filters on samples from Denisova Cave, we assigned each detected family a label based on the likelihood of its DNA being present in the cave sediments of Denisova Cave: ‘Evidence’ if fossil evidence for the family exists at Denisova Cave (Vasiliev, Shunkov, and Kozlikin 2013; 2017), ‘Possible’ if the geographical distribution of the family overlaps with Denisova Cave, and ‘Unlikely’ if neither condition was met.

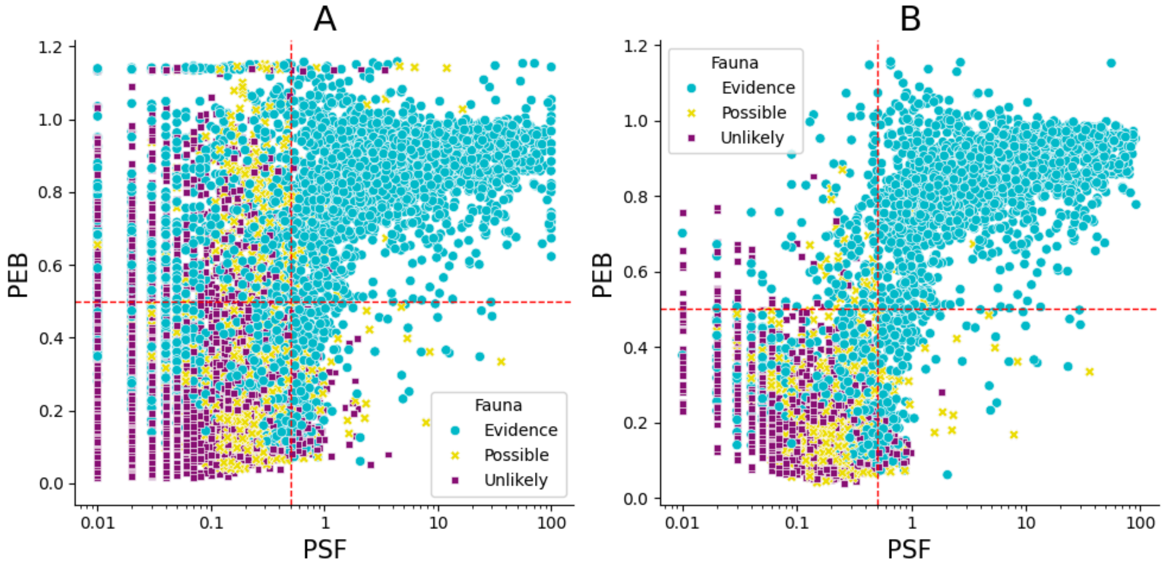

Figure S7.1 | Effects of post-pipeline filters on quicksand results. Each point in the scatterplots represents a mammalian family detected by quicksand v2.3 in one of the 274 mammalian mtDNA capture libraries, colored by the likelihood of its presence in Denisova Cave. Families are stratified by the percentage of unique sequences in the sample (PSF) and the proportion of expected breadth (PEB) in the alignment to the respective “best” reference genome. Red lines indicate the applied filter thresholds; families falling below the horizontal

line and to the left of the vertical line are removed from the results. (A) All family assignments. (B) Only families labeled as ancient (++) by quicksand.

The majority of “unlikely” or “possible” family classifications were assigned a PSF below 0.5% or a PEB below 0.5, matching the patterns of false positive assignments observed in the simulated datasets. As shown in Fig. S7.2, applying these filters results in only ten ancient families consistently meeting both thresholds, while additional seven families met both thresholds in some samples. Among the families meeting both thresholds, all (but one with a “Possible” label) carry the “Evidence” label, suggesting reliable assignments and successful filtering.

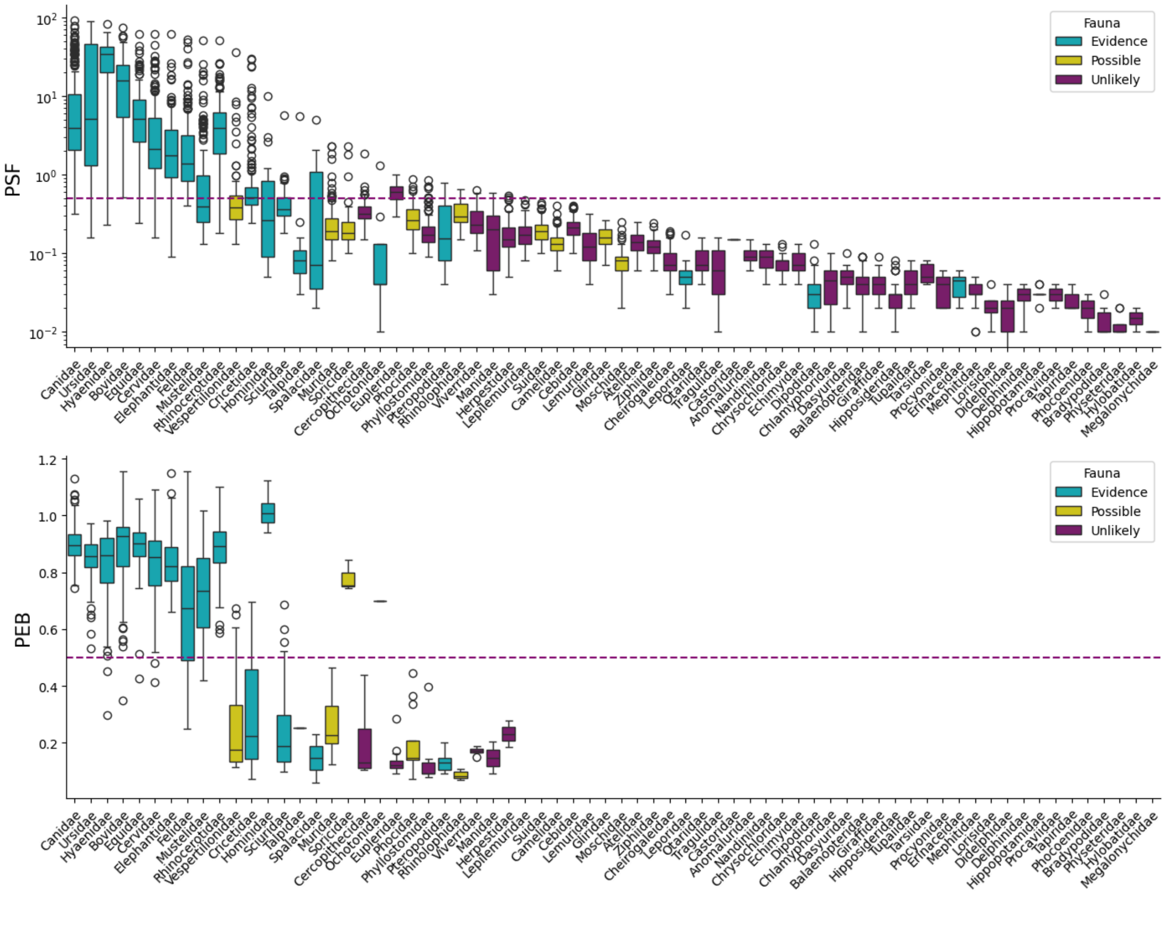

Figure S7.2 | Filtering mammalian mtDNA enrichment samples.
Both plots show the filtering steps for “ancient” families (++). **Upper panel:** Distribution of PSF values across all 274 samples and families. Box colors correspond to the likelihood classification of each family. The red horizontal line indicates the 0.5% PSF cutoff. **Lower plot:** Distribution of PEB values for the same family assignments that passed the 0.5% sequence cutoff. The red horizontal line indicates the 0.5 PEB cutoff. Boxplot formatting follows Fig. 2.

After applying both filters, we found ancient mammalian mtDNA in 271 of the 274 sediment samples (Zavala et al. 2021: 167) and were able to detect additional ancient families in 205

samples that were not reported by Zavala et al. 2021. 44 samples showed fewer ancient families compared to Zavala et al. 2021 (Table S7.1).

Table S7.1
Number of samples with evidence for ancient DNA (++) of the listed mammalian families in both or either of the quicksand v2.3 and the Zavala et al. 2021 analyses.

Family	both	only quicksand	only Zavala et al. 2021
Canidae	227	36	
Ursidae	207	43	
Bovidae	201	37	2
Rhinocerotidae	175	34	3
Equidae	169	14	3
Cervidae	136	56	3
Hyaenidae	134	58	1
Felidae	125	60	15
Elephantidae	112	59	
Mustelidae	45	23	3
Hominidae	4	6	
Cricetidae	1	16	13
Vespertilionidae	0	5	4
Talpidae	0	0	1
Muridae	0	0	1

7.2 Lineage Assignment of the human mtDNA capture libraries

For all positive samples we performed a lineage assignment test as described in Meyer et al. 2016. The lineages for this test included Neanderthals, Hohlenstein-Stadel Neanderthals, the Sima de los Huesos hominins, Denisovans, the shared Denisova-Sima lineage, and modern humans. In brief, we counted for each sample the number of diagnostic positions in the rCRS genome overlapped by aligned mtDNA fragments and the number of positions where the fragments show the lineage-specific (‘diagnostic’) state for each lineage. The diagnostic positions for these lineages were taken from Zavala et al. 2021 and created by comparing multiple hominin genomes, selecting the sites where 99% of the genomes of one lineage differed from all others (“nochimp0.99”, see Zavala et al. 2021: Supplementary Data File 3 for the used genomes). The support for each lineage was calculated as the percent of shared

states among all overlapping diagnostic positions. Lineage assignments were retained if the lineage support exceeded 10% and at least three different shared diagnostic positions were identified. Diagnostic positions that were overrepresented in the dataset (i.e., present in more samples than two standard deviations above the mean number of samples per diagnostic position, see Zavala et al. 2021; SI 4.1) were removed and assignments to the modern human lineage were retained if they could be confirmed using only deaminated mtDNA fragments to reduce the impact of modern human contamination.

Supplementary Section 8: Evaluating quicksand for the analysis of shotgun and capture data from different library types

quicksand was developed and validated for the analysis of mammalian and human mitochondrial DNA (mtDNA) in libraries enriched for mammalian or human mtDNA. It can *in principle* be applied to shotgun sequencing data as well. However, in environmental shotgun datasets, the proportion of total mammalian DNA (nuclear plus mitochondrial) is usually below 1% (e.g. Ottoni et al. 2019; Gelabert et al. 2021). As mitochondrial DNA makes up only about 0.03-0.3% of the total genomic material in a cell (In humans: ~100-1,000 copies of a ~17 kb genome compared to two copies of a 3 Gb nuclear genome), a shotgun dataset with 10 million sequences, by rule of thumb, yields only ~30-300 mammalian mtDNA sequences. As a consequence, the likelihood of detecting mammalian mtDNA with quicksand strongly depends on the ancient DNA preservation in the sample and the sequencing depth (see SI 8.2.3 and SI 8.3.2).

In this supplementary section, we provide examples for the analysis of DNA-libraries published in three different studies to demonstrate the use of quicksand on different library- (single- and double-stranded) and experiment types (capture and shotgun). We show how the final summary report is evaluated and how filter thresholds can be adjusted.

The columns used to evaluate the final summary report are 'FamPercentage' and 'ProportionExpectedBreadth', which correspond to the PSF and PEB filter values, respectively (see SI4). Importantly, the PEB filter is generally more informative than the PSF filter because the breadth of coverage is a key metric for determining assignment accuracy. However, since PEB assumes unbiased (random) mapping of sequences to the reference genome, lower values may occur when this expectation is violated; for instance, due to capture or genetic divergence from the selected 'best' reference genome in the database (See SI 8.2.2).
Additional columns provided in the report for quality control include the KrakenUniq statistics: 'Kmers' (unique kmer count), 'KmerCoverage' (proportion of kmers observed), and 'KmerDupRate' (average number of times each kmer was observed) as well as the 'ProportionMapped' column. Filters are not applied to these metrics but they can be useful for troubleshooting (see SI 8.3.2).

8.1 Download quicksand-database

Runtime: ~4-5h

To follow the examples in this section, please download the quicksand-database available on the MPI-EVA FTP servers or construct a custom version of the database using the quicksand-build pipeline (https://github.com/mpieva/quicksand-build).

To download the full reference database (~60GB) run:

```
latest=$(curl http://ftp.eva.mpg.de/quicksand/LATEST)

wget -r -np -nc -nH --cut-dirs=3 \
     --reject="*index.html*" -q --show-progress \
     -P refseq \
     http://ftp.eva.mpg.de/quicksand/build/$latest
```

quicksand was developed and tested using a database built from the *mammalian* mtDNA genomes in RefSeq. For broader usability, the database provided on the FTP server was built from *full* RefSeq, including non-mammalian mtDNA genomes. This was done, because database construction is computationally intensive and may not be feasible for users with limited computational resources. While this expanded database enables the detection of a wider range of organisms, quicksand (and the filter thresholds) have not been systematically tested for the validation of the non-mammalian assignments. For this section, we therefore focus on the mammalian classifications only.

8.2 Single stranded Libraries

For the estimation of deamination rates quicksand by default assumes that sequences are derived from libraries prepared using a single stranded library preparation protocol (ssDNA). For the analysis of libraries prepared with a double stranded library preparation method, see section SI8.3

First, we demonstrate the evaluation of quicksand for ssDNA libraries enriched for mammalian mtDNA (SI 8.2.1) and human mtDNA (SI 8.2.2) as well as shallow shotgun sequencing data (SI 8.2.3). All example libraries come from the same sediment sample (SP3854), collected from Layer 15 of Denisova Cave, East Chamber, and published in Slon et al. 2017. The data are available in the European Nucleotide Archive (ENA), and an overview of the libraries is provided in Table S8.1. For an example of the analysis of *multiple samples* in parallel, see SI 8.3.1.

Table S8.1
List of ssDNA libraries used in this section

Sample	Layer	Library	Library Type	Sequences	ENA Entry
SP3854	EC 15	R4095	Mammalian mtDNA Capture	1,837,960	ERR1883545
SP3854	EC 15	R4051	Human mtDNA Capture	1,512,964	ERR1883655
SP3854	EC 15	R5723	Shotgun	1,638,519	ERR1883446

8.2.1 ssDNA / Mammalian mtDNA Capture

Runtime: ~3 min.

With the quicksand database downloaded to the 'refseq' directory (Section SI8.1), create a subdirectory for the mammalian mtDNA capture analysis and download the required library file into a fresh input directory ('split'):

```
mkdir ssDNA_mammMT && cd ssDNA_mammMT
wget -P split
ftp.sra.ebi.ac.uk/vol1/run/ERR188/ERR1883545/R4095.bam
```

Then run quicksand with the default parameters:

```
nextflow run mpieva/quicksand \
     -r v2.5 \
     --split split/ \
     --db ../refseq/kraken/Mito_db_kmer22/ \
     --genomes ../refseq/genomes/ \
     --bedfiles ../refseq/masked/ \
     -profile singularity
```

After completion, the final summary report is saved as '`quicksand_v2.5/final_report.tsv`'. In total, 22 mammalian families are reported of which 20 show 'Ancientness' levels of + or ++ (Table S8.2). Nine mammalian families pass the default PSF and PEB filter thresholds (see '`quicksand_v2.5/filtered_report_0.5p_0.5b.tsv`').

Table S8.2
Mammalian families detected by quicksand in sample SP3854 (mammalian mtDNA capture library: R4095), sorted by unique/deduplicated sequences.

Family	Best Reference	Reads Deduped	PSF	Coverage	PEB
**Hyaenidae**	Crocuta_crocuta	7616	**34.98**	24.84x	**0.662**
**Rhinocerotidae**	Coelodonta_antiquitatis	3965	**19.15**	11.32x	**0.934**
**Bovidae**	Bos_grunniens	3448	**16.16**	10.17x	**0.824**
**Ursidae**	Ursus_spelaeus	1066	**5.04**	3.26x	**0.757**
**Canidae**	Canis_lupus	970	**4.52**	2.71x	**0.758**
**Equidae**	Equus_ovodovi	884	**4.24**	2.72x	**0.703**
**Elephantidae**	Mammuthus_columbi	553	**2.65**	1.61x	**0.711**
**Mustelidae**	Martes_zibellina	514	**2.45**	1.51x	**0.795**
**Cervidae**	Cervus_nippon	281	**1.32**	0.82x	**0.718**

Felidae	Panthera_tigris	369	**1.74**	1.04x	0.346
Eupleridae	Galidia_elegans	225	**1.10**	0.7x	0.103
Phocidae	Monachus_monachus	166	**0.81**	0.47x	0.131
Sciuridae	Marmota_himalayana	155	**0.75**	0.44x	0.291
Muridae	Rattus_norvegicus	141	**0.67**	0.4x	0.193
Viverridae	Paguma_larvata	112	0.26	0.32x	0.331
Cercopithecidae	Macaca_sylvanus	70	0.33	0.2x	0.23
Cricetidae	Neodon_irene	65	0.31	0.18x	0.443
Cebidae	Sapajus_xanthosternos	64	0.24	0.19x	0.148
Hominidae	Homo_sapiens_subsp._' Denisova'	41	0.19	0.13x	**1.005**
Otariidae	Zalophus_japonicus	33	0.16	0.09x	0.402

Note: The columns 'PSF' and 'PEB' are called 'FamPercentage' and 'ProportionExpectedBreadth' in the final summary report. Shortened here to save space
bold: values that pass the PSF or PEB default filter thresholds
italic: Families with non-significant 'Ancientness' level (+)

The mammalian mtDNA enrichment analyzed here (targeting 242 mammalian mtDNA genomes) generally provides sufficiently random coverage across all mammalian families for the PEB filter to be effective. The lower PEB value observed for the Hyaenidae assignment (0.66), however, is likely due to the absence of Hyaenidae mtDNA in the capture probe set.

The Felidae, Eupleridae, Phocidae, and Sciuridae assignments pass the PSF filter but not the PEB filter. With genomic coverages between 0.4x and 1x and PEB values ranging from 0.1 to 0.34, these cases are likely false positives (see Fig. S4.3). In contrast, the Hominidae assignment passes the PEB filter (PEB = 1.0) but not the PSF filter. However, the high PEB value for the Hominidae, combined with a mean genomic coverage of 0.1x, indicates an authentic assignment that would benefit from additional data for verification (e.g. through human mtDNA capture).

In summary, for generic mammalian mtDNA capture libraries, the default quicksand filters (PSF = 0.5 and PEB = 0.5) provide a reliable but conservative set of family assignments. Filtered families with a high PEB value could be authentic but require additional verification.

8.2.2 ssDNA / Human mtDNA Capture

Runtime: ~3 min.

After downloading the quicksand database to the 'refseq' directory (Section SI8.1), create a subdirectory for the human mtDNA capture analysis. Then, download the required library file into a new input directory ('split'):

```
mkdir ssDNA_humMT && cd ssDNA_humMT
wget -P split
ftp.sra.ebi.ac.uk/vol1/run/ERR188/ERR1883655/R4051.bam
```

Next, run quicksand using the default parameters:

```
nextflow run mpieva/quicksand \
     -r v2.5 \
     --split split/ \
     --db ../refseq/kraken/Mito_db_kmer22/ \
     --genomes ../refseq/genomes/ \
     --bedfiles ../refseq/masked/ \
     -profile singularity
```

After completion, the final summary report is saved as '`quicksand_v2.5/final_report.tsv`'. A total of 15 mammalian families are detected (Table S8.3). Only the Hominidae family passes the default PSF and PEB filter thresholds (see '`quicksand_v2.5/filtered_report_0.5p_0.5b.tsv`').

Table S8.3
All mammalian families detected by quicksand in sample SP3854 (human mtDNA capture library: R4051), sorted by unique/deduplicated sequences.

Family	Best Reference	Reads Deduped	PSF	Coverage	PEB
Hyaenidae	Crocuta_crocuta	2894	**55.97**	9.75x	0.19
Rhinocerotidae	Coelodonta_antiquitatis	587	**12.61**	1.94x	0.192
Bovidae	Bos_taurus	280	**5.40**	0.86x	0.231
Equidae	Equus_ovodovi	154	**3.24**	0.5x	0.252
Felidae	Neofelis_diardi	146	**2.81**	0.43x	0.107
Sciuridae	Dremomys_rufigenis	132	**2.60**	0.35x	0.19
**Hominidae**	**Homo_sapiens_subsp._'Denisova'**	**118**	**2.28**	**0.4x**	**0.968**
Cervidae	Cervus_hanglu	102	**2.17**	0.3x	0.202
Muridae	Rattus_norvegicus	99	**2.10**	0.29x	0.171

Cercopithecidae	Macaca_nigra	85	**1.80**	0.27x	0.152
Ursidae	Ursus_spelaeus	81	**1.44**	0.27x	0.354
Mustelidae	Martes_zibellina	75	**1.50**	0.25x	0.314
Canidae	Canis_aureus	64	**1.35**	0.2x	0.312
Elephantidae	Mammuthus_primigenius	55	**1.18**	0.15x	0.322
Cricetidae	Phodopus_sungorus	36	**0.77**	0.09x	0.351

Note: Column 'PSF' and 'PEB' are called 'FamPercentage' and 'ProportionExpectedBreadth' in the quicksand summary report. Shortened here to save space
bold: Families that pass both the PSF and PEB default filter thresholds
italic: Non ancient assignments

Even in the data from the human mtDNA capture, assignments are dominated by faunal "bycatch," i.e., mtDNA from other mammalian families that is also recovered by the human mtDNA probes. However, unlike for the mammalian mtDNA capture (SI 8.2.1), non-human assignments no longer meet the expectation of random mapping because they are biased towards the subset of sequences similar to the human mtDNA probes. This substantially lowers the PEB-values of all non-human assignments, which range from 0.1 to 0.35 (Table S8.3). As a consequence, the PEB filter *cannot* be used to securely identify true-positive assignments among the faunal bycatch; it can do so only for the Hominidae, the only family that meets the criterion of random mapping.

In addition, the PSF-values in human mtDNA captures are less informative for distinguishing true- from false-positive families. The above-mentioned capture bias, combined with a reduced number of total detected families, changes the distribution of PSF values compared to the mammalian capture. We find an increased PSF for the dominant family (e.g., Hyaenidae: 55.97 vs. 34.98) and a leveling of PSF values across the remaining families. In this example, all mammalian families pass the default PSF threshold (Table S8.3).

Because the human mtDNA capture experiment is intentionally biased toward Hominidae, applying the PSF filter is optional, as it could remove the sequences we are specifically trying to detect. Instead, we recommend relying on the PEB filter to assess the quality of Hominidae assignments. For a more conservative approach, a stricter PEB threshold (e.g., 0.7) combined with a minimum number of reads or genomic coverage (e.g., >0.1x) could be applied.

8.2.3 ssDNA / Shotgun Sequencing

Runtime: ~2 min.

After downloading the quicksand database to the ‘refseq’ directory (Section SI8.1), create a subdirectory for the shotgun library analysis. Then, download the required library file into a new input directory (‘split’):

```
mkdir ssDNA_shotgun && cd ssDNA_shotgun

wget -P split \
     ftp.sra.ebi.ac.uk/vol1/run/ERR188/ERR1883446/R5723.bam
```

Next, run quicksand using the default parameters:

```
nextflow run mpieva/quicksand \
     -r v2.5 \
     --split split/ \
     --db ../refseq/kraken/Mito_db_kmer22/ \
     --genomes ../refseq/genomes/ \
     --bedfiles ../refseq/masked/ \
     -profile singularity
```

After completion, the final summary report is saved as ‘`quicksand_v2.5/final_report.tsv`’. A single mammalian family, the Hyaenidea is detected with 33 sequences (Table S8.4), showing an Ancientness level of ++ and passing both the default PSF and PEB filter thresholds (‘`quicksand_v2.5/filtered_report_0.5p_0.5b.tsv`’).

Table S8.4
Hyaenidae assignment detected by quicksand in sample SP3854 (shotgun library R5723).

Family	Best Reference	Reads Deduped	PSF	Coverage	PEB
Hyaenidae	Crocuta_crocuta	33	40.50	0.08x	1.153

With the low sequencing depth (1,638,519 sequences), only the Hyaenidae are detected by quicksand. The assignments in the shotgun data are unaffected by capture bias, resulting in a PEB value of ~1 for the Hyaenidae.

As outlined in SI 4.1, the final summary report includes only assignments that pass the initial *KrakenUniq* filters. As we demonstrated with simulated data (SI 6.1), the default thresholds for the number of unique kmers can be lowered for a more sensitive screening, allowing additional families to pass the initial KrakenUniq step and proceed to mapping and downstream evaluation.

quicksand saves the KrakenUniq report for each sample, which can be examined to identify families that would be included if the filter thresholds were lowered. In this shotgun library, the report (‘`quicksand_v2.5/stats/R5723.kraken.report`’) lists 10 mammalian family assignments, of which only the Hyaenidae meets the default thresholds of 129 unique kmers and 3 sequences (Table S8.5).

Table S8.5
Overview of all detected mammalian families in the KrakenUniq report (`` `quicksand_v2.5/stats/R5723.kraken.report` ``), sorted by number of unique kmers

TaxReads	Kmers	Rank	Name
**39**	**317**	**family**	**Hyaenidae**
**16**	82	family	Bovidae
**6**	48	family	Rhinocerotidae
**15**	30	family	Muridae
**10**	29	family	Vespertilionidae
**15**	24	family	Cricetidae
**7**	16	family	Pteropodidae
**6**	12	family	Soricidae
2	11	family	Otariidae
2	11	family	Ursidae

Bold: Families passing the default filter of 129 unique kmers and 3 sequences per family

To override the default filters and process all potential mammalian families, repeat the quicksand-run with the flags `--krakenuniq_min_kmers 11` and `--krakenuniq_min_reads 2` to apply more permissive KrakenUniq filter thresholds.

```
# rename the folder, to not overwrite it
mv quicksand_v2.5/ quicksand_v2.5.old/

nextflow run mpieva/quicksand \
     -r v2.5 \
     --split split/ \
     --db ../refseq/kraken/Mito_db_kmer22/ \
     --genomes ../refseq/genomes/ \
     --bedfiles ../refseq/masked/ \
     --krakenuniq_min_kmers 11 \
     --krakenuniq_min_reads 2 \
     -profile singularity
```

The lower filter thresholds increase the runtime (~10 minutes) and allow 600 families to pass the KrakenUniq step. Of these, only 62 assignments have at least one mapped sequence, and 57 pass the PEB and PSF filters. Only four mammalian families appear in the final summary report and pass both filters (Table S8.6). Given the low sequence counts per family, only the Hyaenidae shows significant Ancientness levels (++). Although the number of sequences is very low, the detected families correspond to the four most abundant in the sample, as determined using the mammalian capture library (Table S8.2).

Table S8.6
quicksand results for mammalian assignments (final report) for sample SP3854 (shotgun library R5723), reduced kmer-filters. sorted by unique sequences.

Family	Best Reference	Reads Deduped	PSF	Coverage	PEB
Hyaenidae (++)	Crocuta_crocuta	33	17.58	0.085x	1.153
Rhinocerotidae	Coelodonta_antiquitatis	5	2.74	0.01x	1.1
Bovidae	Procapra_przewalskii	2	1.09	0.0x	1.135
Ursidae	Ursus_spelaeus	1	0.54	0.0x	1.134

Note: This table contains only assignments with at least 1 mapped sequence after KrakenUniq assignment. Column ‘PSF’ and ‘PEB’ are called ‘FamPercentage’ and ‘ProportionExpectedBreadth’ in the quicksand summary report. Shortened here to save space

To summarize, while we discourage using quicksand on shallow shotgun sequencing data to draw biological conclusions about the taxonomic composition of a sediment sample, it can provide a first impression of DNA preservation and help prioritize samples for follow-up capture experiments.

In contrast, Section 8.3.2 shows the application of quicksand on a deeply sequenced double-stranded library.

8.3 Double stranded libraries

Sequences from libraries prepared with a double-stranded (dsDNA) protocol display different deamination patterns compared to those from single-stranded libraries, showing G-to-A substitutions at the 3′ ends of DNA sequences rather than the C-to-T substitutions typical of single-stranded libraries. To account for these G-to-A patterns in the Ancientness assessment, quicksand needs to be run with the `--doublestranded` flag

8.3.1 dsDNA / Mammalian mtDNA Capture

Runtime: ~1.5h

For the analysis of double-stranded mammalian mitochondrial capture libraries, we used published data from sediment samples collected at El Mirón Cave (Cantabria, Spain). These data were generated by Gelabert et al. 2025 and are available in the ENA under the accession ID PRJEB74514 (Table S8.6). The libraries were enriched for 51 mammalian mtDNA genomes (Tejero et al. 2024) and sequenced to a depth of 5–10 million reads per library. In the original study, Gelabert et al. analyzed the data using *euka* to bin sequences and assess aDNA preservation, followed by a BLAST/MEGAN-based approach to refine taxonomic classifications to the genus and species levels.

Table S8.6
List of El Mirón sediment samples in the ENA (accession PRJEB74514) with their associated stratigraphic layers and sample numbers.

ENA Entry	Sample	Layer	ENA Entry	Sample	Layer
ERR13916465	1	121	ERR13916459	16	122/124
ERR13916464	2	122/124	ERR13916456	17	125
ERR13916460	3	122/124	ERR13916454	18	126
ERR13916458	4	122/124	ERR13916450	19	127
ERR13916455	5	125	ERR13916448	20	128
ERR13916453	6	126	ERR13916440	21	129
ERR13916452	7	127	ERR13916436	22	130
ERR13916449	8	128	ERR13916433	23	130
ERR13916439	9	129	ERR13916430	24	130
ERR13916429	10	130	ERR13916467	26	119
ERR13916432	11	130	ERR13916443	28	128
ERR13916435	12	130	ERR13916446	30	128

ERR13916466	13	121	ERR13916445	32	128
ERR13916462	14	122/124	ERR13916442	34	128
ERR13916461	15	122/124	ERR13916438	36	130
			ERR13916437	38	130

After downloading the quicksand database to the ‘refseq’ directory (Section SI8.1), create a subdirectory for the analysis:

```
mkdir dsDNA_miron && cd dsDNA_miron
```

Then, download the required 32 library files into a new input directory (‘split’) for parallel processing:

```
mkdir split && cd split \
wget -nc ftp.sra.ebi.ac.uk/vol1/err/ERR139/038/ERR13916438/ERR13916438.bam
wget -nc ftp.sra.ebi.ac.uk/vol1/err/ERR139/067/ERR13916467/ERR13916467.bam
wget -nc ftp.sra.ebi.ac.uk/vol1/err/ERR139/037/ERR13916437/ERR13916437.bam
wget -nc ftp.sra.ebi.ac.uk/vol1/err/ERR139/066/ERR13916466/ERR13916466.bam
wget -nc ftp.sra.ebi.ac.uk/vol1/err/ERR139/082/ERR13953082/ERR13953082.bam
wget -nc ftp.sra.ebi.ac.uk/vol1/err/ERR139/043/ERR13916443/ERR13916443.bam
wget -nc ftp.sra.ebi.ac.uk/vol1/err/ERR139/046/ERR13916446/ERR13916446.bam
wget -nc ftp.sra.ebi.ac.uk/vol1/err/ERR139/059/ERR13916459/ERR13916459.bam
wget -nc ftp.sra.ebi.ac.uk/vol1/err/ERR139/035/ERR13916435/ERR13916435.bam
wget -nc ftp.sra.ebi.ac.uk/vol1/err/ERR139/040/ERR13916440/ERR13916440.bam
wget -nc ftp.sra.ebi.ac.uk/vol1/err/ERR139/052/ERR13916452/ERR13916452.bam
wget -nc ftp.sra.ebi.ac.uk/vol1/err/ERR139/045/ERR13916445/ERR13916445.bam
wget -nc ftp.sra.ebi.ac.uk/vol1/err/ERR139/030/ERR13916430/ERR13916430.bam
wget -nc ftp.sra.ebi.ac.uk/vol1/err/ERR139/062/ERR13916462/ERR13916462.bam
wget -nc ftp.sra.ebi.ac.uk/vol1/err/ERR139/053/ERR13916453/ERR13916453.bam
wget -nc ftp.sra.ebi.ac.uk/vol1/err/ERR139/036/ERR13916436/ERR13916436.bam
wget -nc ftp.sra.ebi.ac.uk/vol1/err/ERR139/032/ERR13916432/ERR13916432.bam
wget -nc ftp.sra.ebi.ac.uk/vol1/err/ERR139/056/ERR13916456/ERR13916456.bam
wget -nc ftp.sra.ebi.ac.uk/vol1/err/ERR139/061/ERR13916461/ERR13916461.bam
wget -nc ftp.sra.ebi.ac.uk/vol1/err/ERR139/054/ERR13916454/ERR13916454.bam
wget -nc ftp.sra.ebi.ac.uk/vol1/err/ERR139/049/ERR13916449/ERR13916449.bam
wget -nc ftp.sra.ebi.ac.uk/vol1/err/ERR139/058/ERR13916458/ERR13916458.bam
wget -nc ftp.sra.ebi.ac.uk/vol1/err/ERR139/055/ERR13916455/ERR13916455.bam
wget -nc ftp.sra.ebi.ac.uk/vol1/err/ERR139/060/ERR13916460/ERR13916460.bam
wget -nc ftp.sra.ebi.ac.uk/vol1/err/ERR139/029/ERR13916429/ERR13916429.bam
wget -nc ftp.sra.ebi.ac.uk/vol1/err/ERR139/065/ERR13916465/ERR13916465.bam
wget -nc ftp.sra.ebi.ac.uk/vol1/err/ERR139/064/ERR13916464/ERR13916464.bam
wget -nc ftp.sra.ebi.ac.uk/vol1/err/ERR139/042/ERR13916442/ERR13916442.bam
wget -nc ftp.sra.ebi.ac.uk/vol1/err/ERR139/033/ERR13916433/ERR13916433.bam
wget -nc ftp.sra.ebi.ac.uk/vol1/err/ERR139/048/ERR13916448/ERR13916448.bam
wget -nc ftp.sra.ebi.ac.uk/vol1/err/ERR139/050/ERR13916450/ERR13916450.bam
wget -nc ftp.sra.ebi.ac.uk/vol1/err/ERR139/039/ERR13916439/ERR13916439.bam

cd ../
```

Next, run quicksand using the default parameters and the `--doublestranded` flag:

```
quicksand run mpieva/quicksand \
     -r v2.5 \
     --split split \
     --genomes  refseq/genomes \
     --db refseq/kraken/Mito_db_kmer22 \
     --bedfiles refseq/masked \
     --doublestranded \
     -profile singularity
```

After completion, the summary report contains the results for all 32 libraries. Applying the default filters (PSF of 0.5 and PEB of 0.5; '`quicksand_v2.5/filtered_report_0.5p_0.5b.tsv`'), recovers the same mammalian taxa reported by Gelabert et al. (Table S8.7). Interestingly, we also detected ancient DNA from several bird families across multiple samples: Accipitridae (11 samples), Columbidae (6), Falconidae (2), Phasianidae (3), and Strigidae (11), as well as ancient fish mtDNA (Salmonidae) in 14 samples, consistent with archaeological evidence for these families in El Mirón Cave (Demarchi et al. 2019; Consuegra et al. 2002). All these families pass the PEB-filter, even though they are not part of the mammalian enrichment set (Tejero et al. 2024; supplementary data).

Table S8.7
Summary of quicksand results showing the detected ancient mammalian families

Ancient Family	Best References (genera)	Number of Samples (Samples with > 5x Coverage)	Layers
Bovidae	Capra, Bos	31 (26)	**All**
Canidae	Canis, Cuon, Vulpes	32 (23)	**All**
Cervidae	Capreolus, Cervus, Rangifer	32 (28)	**All**
Cricetidae	Microtus	28 (15)	**121**, **122/124**, **125**, 126, **127**, **128**, **129**, **130**
Elephantidae	Mammuthus	4 (0)	128,130
Equidae	Equus	22 (3)	121, 122/124, **125**, **126**, 127, 128, **129**, 130
Felidae	Lynx, Panthera, Felis	24 (3)	119, 122/124, **125**, 126, 127, **128**, **129**, 130
Hominidae	Homo	13 (2)	121, **122/124**, 125, **126**, 127, 129
Hyaenidae	Crocuta	19 (9)	**119**, 121, 125, **126**, **128**, **129**, **130**
Leporidae	Lepus	13 (0)	121, 122/124, 125, 126, 127, 128, 129
Muridae	Apodemus	5 (0)	130
Mustelidae	Mustela	2 (2)	**122/124, 128**
Rhinocerotidae	Coelodonta	3 (0)	130
Soricidae	Sorex	7 (2)	119, **127**, **128**, 130
Suidae	Sus	3 (0)	130
Talpidae	Talpa	13 (0)	121, 122/124, 126, 127, 128, 130
Ursidae	Ursus	24 (2)	119, 122/124, 125, **126**, 127, 128, **129**, 130

Note: The bold layers in the 'Layers' column indicate where samples with >5x coverage were found.

The original study reported 70 assignments with coverage >5x. Re-analysis of the data using quicksand identifies more samples exceeding this threshold (115) and with higher coverage for all reported families.

To illustrate how quicksand output can be analysed further, we here repeat the human haplogroup analysis for three samples from the Solutrean levels 121, 122, and 126 reported by Gelabert et al. 2025. In all three cases, quicksand yields higher mtDNA coverage than previously reported: 4.86x, 10.71x, and 11.37x compared to 2.5x, 5.6x, and 6.4x for levels 121, 122, and 126, respectively.

Haplogroup calling with HaploGrep 3 (Schönherr et al., 2023) requires an mtDNA consensus sequence based on alignment to the rCRS. As shown in Tables S8.2 and S8.3, however, the "best" reference genome for the Hominidae family in default quicksand runs is often the Denisovan or Neanderthal mtDNA genome. This occurs because Neanderthals and Denisovans are classified as subspecies of modern humans in the NCBI taxonomy (https://www.ncbi.nlm.nih.gov/Taxonomy/Browser/wwwtax.cgi?command=show&mode=tree&id=9606). Consequently, if KrakenUniq matches kmers to both the modern human and Neanderthal/Denisovan mtDNA genomes, the 'best' assignment is placed at the subspecies (Neanderthal/Denisovan) level rather than at the *Homo* node.

This issue can be avoided by running quicksand with a set of predefined ("fixed") reference genomes for selected families, either directly or in a second step using the `--rerun` flag. In "rerun" mode, quicksand skips the preprocessing and KrakenUniq classification and starts from the ExtractedReads for the selected "fixed" families (e.g., '`quicksand_v2.5/out/Hominidae/1-extracted/*.bam`'). quicksand then processes these sequences using the fixed reference genomes and updates the final summary reports to include the statistics for the additional mappings. This example shows the combined use of the `--fixed` with the `--rerun` flag.

The input for the `--fixed` flag is a tab-separated file specifying the family, a unique tag (or species name), and the absolute path to the mtDNA reference genome to be used. The number of fixed references per family is not limited, allowing alignments and statistics to be generated for multiple reference genomes per family if needed.

Use quicksand with the `--rerun` and `--fixed` options to process all Hominidae sequences (from the first run) with the rCRS. The rCRS is the 'Homo_sapiens' reference mtDNA genome that is part of the quicksand database:

1. Prepare the ‘fixed.tsv’ file:

```
export FAMILY=”Hominidae”
export TAG=”rCRS”
export
GENOME=”$PWD/../refseq/genomes/Hominidae/Homo_sapiens.fasta”

echo -e "Family\tTag\tGenome" > fixed.tsv
echo -e $FAMILY\t$TAG\t&GENOME >> fixed.tsv
```

2. Repeat the quicksand run with the `--rerun` and `--fixed` options, as well as the `--doublestranded` flag:

```
quicksand run mpieva/quicksand \
     -r v2.5 \
     --fixed fixed.tsv \
     --rerun \
     --doublestranded \
     -profile singularity
```

The mapped and deduplicated bam-files can be found in the freshly created ‘fixed’ subdirectory of the family specific output-folder (‘`quicksand_v2.5/out/Hominidae/fixed/3-deduped/`’)

Next, construct a FASTA consensus sequence from the rCRS-mapped and deduplicated sequences of each sample using ANGSD v0.940 (Korneliussen et al., 2014), with the most common allele option (`-doFasta 2` and `-doCounts 1`) and trimming the first and last base of each sequence to mitigate aDNA damage effects (`-trim 1`).

1. index the sample BAM-files:

```
samtools index
quicksand_v2.5/out/Hominidae/fixed/3-deduped/ERR13916465.Hominidae
.rCRS_deduped.bam

samtools index
quicksand_v2.5/out/Hominidae/fixed/3-deduped/ERR13916462.Hominidae
.rCRS_deduped.bam

samtools index
quicksand_v2.5/out/Hominidae/fixed/3-deduped/ERR13916454.Hominidae
.rCRS_deduped.bam
```

2. Create FASTA consensus sequences:

```
angsd -out ERR13916465.fasta -i
quicksand_v2.5/out/Hominidae/fixed/3-deduped/ERR13916465.Hominidae
.rCRS_deduped.bam -doFasta 2 -doCounts 1 -trim 1

angsd -out ERR13916462.fasta -i
quicksand_v2.5/out/Hominidae/fixed/3-deduped/ERR13916462.Hominidae
.rCRS_deduped.bam -doFasta 2 -doCounts 1 -trim 1

angsd -out ERR13916454.fasta -i
quicksand_v2.5/out/Hominidae/fixed/3-deduped/ERR13916454.Hominidae
.rCRS_deduped.bam -doFasta 2 -doCounts 1 -trim 1
```

Next, use the HaploGrep 3 web tool (https://haplogrep.i-med.ac.at/) to assign human mtDNA haplogroups to each sample (Table S8.8).

Table S8.8
Comparison of the haplogroups obtained by Gelabert et al. (2015) with those from the quicksand results

Sample	Layer	Gelabert et al. 2015	This study
ERR13916465	121	R or U	U
ERR13916462	122	U2'3'4'7'8'9	U2'3'4'7'8'9
ERR13916454	126	U2'3'4'7'8'9	U4’9

The detected haplogroups are consistent with those reported by Gelabert et al. 2025. Due to the increased amount of data recovered using quicksand, we were able to refine the haplogroup assignments for sample ERR13916465 (U) and ERR13916454 (U4’9, a clade within U2'3'4'7'8'9).

While a complete re-evaluation of this dataset is beyond the scope of this example, this chapter demonstrates that quicksand can be used to analyze mammalian capture enrichment data generated from double-stranded DNA libraries using a different probeset. The results are consistent with those obtained in the original publication, and quicksand classifies more sequences, allowing the identification of additional families and providing more data for mtDNA haplogroup assignment.

We also illustrate how quicksand output can be used for additional downstream analyses and how run options can be adjusted to meet the requirements of external tools. While Gelabert et al. applied several additional quality-control and filtering steps to remove modern faunal contaminants (not replicated here), we show that quicksand is suitable to provide a quick and easy starting point for subsequent in-depth, taxa-specific analyses.

8.3.2 dsDNA / Shotgun Sequencing

Runtime: ~4h

This chapter demonstrates the analysis of a deeply sequenced dsDNA shotgun library with quicksand. For this example, we use data from sediment sample 'SAT29' from Satsurblia Cave (Georgia), described in Gelabert et al. 2021, containing ~522 million sequences. The BAM file available from the ENA includes raw reads processed with cutadapt v2.7 to remove sequencing adapters and poly-A–tailed reads. In their screening of the shotgun data, Gelabert et al. report that 1.3% of the sequences are of eukaryotic origin, 44.6% of which from mammalian taxa.

After downloading the quicksand database to the 'refseq' directory (Section SI8.1), create a subdirectory for the shotgun library analysis. Then, download the required library file into a new input directory ('split'):

```
mkdir dsDNA_sat && cd dsDNA_sat

wget -P split \
     ftp://ftp.sra.ebi.ac.uk/vol1/err/ERR602/004/ERR6024164.bam
```

Next, run quicksand using the default parameters and the `--doublestranded` flag:

```
quicksand run mpieva/quicksand \
     -r v2.5 \
     --split split \
     --genomes  ../refseq/genomes \
     --db ../refseq/kraken/Mito_db_kmer22 \
     --bedfiles ../refseq/masked \
     --doublestranded \
     -profile singularity
```

After completion, the final summary report is saved as `'quicksand_v2.5/final_report.tsv'`. quicksand detects 1,237 families, of which 382 have at least one mapped sequence, and 14 pass the PSF and PEB filter thresholds. Five mammalian families are detected: Canidae (best reference: *Canis lupus*), Bovidae (best reference: *Bison bonasus*), Hominidae (best reference: *Homo sapiens subsp. 'Denisova'*), and Cervidae (best reference: *Cervus canadensis*), with Ancientness ratings of ++ and mtDNA coverages of 2.96x, 1.84x, 1.94x, and 0.37x, respectively. In addition, Cricetidae (best reference: *Microtus ilaeus*) is detected with Ancientness + and mtDNA coverage of 0.2x (Table S8.9).

Table S8.9
Mammalian family assignments from sample 'SAT29' (dsDNA shotgun library)

Family	Reads Extracted	Kmers	KmerDupRate	Best Reference
Canidae	2223	101 (8925)	1.69 (2.63)	Canis_lupus_familiaris
Bovidae	1783	1224 (6616)	2.69 (2.33)	Bison_bonasus
Cricetidae	259238	253 (1845)	1.38 (613.0)	Microtus_ilaeus
Hominidae	1080	27 (7082)	1.3 (2.35)	Homo_sapiens_subsp. _'Denisova'
Cervidae	455	58 (1920)	2.21 (1.58)	Cervus_canadensis

Reads Mapped	Reads Deduped	Ancientness	PSF	Coverage	PEB
1666	1130	++	12.42	2.96	0.950
839	570	++	6.18	1.84	0.989
99	66	+	0.71	0.21	1.093
911	644	++	7.10	1.94	1.001
191	133	++	1.51	0.37	1.081

Note: Column 'PSF' and 'PEB' are called 'FamPercentage' and 'ProportionExpectedBreadth' in the quicksand summary report. Shortened here to save space. The 'Kmers' and 'KmerDupRate' columns display the number of unique kmers and the kmer duplication rate for the best reference and, in parentheses, the family level.

We use this example to illustrate how the 'Kmers' and 'KmerDupRate' columns in the final summary report can be used for extended trouble-shooting. As shown in Table S8.9, the Cricetidae stands out with 259,238 sequences classified by KrakenUniq (ReadsExtracted). However, these sequences are based on only 1,845 unique kmers and exhibit a very high kmer duplication rate compared to the other families (613). Particularly telling is that only 99 of the 259,238 sequences successfully map to the best reference genome (ReadsMapped). This combination of KrakenUniq kmer statistics indicates that sequences in the dataset containing a highly repetitive motif are being assigned to Cricetidae, potentially due to a contaminated reference genome in the database or sequence artifacts. Visual inspection of the sequences assigned to Cricetidae ('`quicksand_v2.5/out/Cricetidae/1-extracted/ERR6024164_extractedReads-Cricetidae.bam`') shows that most classified sequences are identical in length (96 bp) and end with the same or highly similar sequence followed by a poly-G tail (ACTCCAGTCACCAGGAGGATCTCGTATGCCGTCTTCTGCTTGAAAA–PolyG), consistent

with technical artifacts such as non-adapter-clipped reads. Although the 99 mapped Cricetidae sequences pass the PSF and PEB quicksand filters and appear authentic, the family-level assignment should be interpreted with caution. We note that Gelabert et al. (2021) used only 226,880,778 sequences for their screening, after applying additional quality-filtering steps that are not replicated here, potentially removing these sequences upfront.

The assignments of the Bovidae, Canidae, and Hominidae families are consistent with the analysis by Gelabert et al. 2021, who used CENTRIFUGE in combination with the whole non-redundant NCBI nucleotide database and a 2% classified-reads cutoff to screen the shotgun data to decide on organisms for follow-up targeted capture experiments and whole genome mapping. In this example, we show that quicksand could be used to replace the CENTRIFUGE step for the detection of mammalian families in the sample. quicksand detects an additional family, the Cervidae, for which archaeological evidence was found in Satsurblia Cave (e.g. Tejero et al. 2024).

In summary, this chapter demonstrates that quicksand can be run with default parameters on deeply sequenced shotgun data to obtain results for the mammalian component that are consistent with the screening of the same data using the complete non-redundant (nuclear) NCBI nucleotide database, as performed by Gelabert et al. 2021. We also show how the KrakenUniq statistics in the final summary report can assist in identifying anomalies in the data and for troubleshooting unexpected assignments.

8.4 Output Structure

Here we outline the structure of the quicksand output. The content of that directory is explained in detail in the documentation (https://quicksand.readthedocs.io/en/latest/in_and_out.html). quicksand output is written to a directory called '`quicksand_{VERSION}`'. The structure of this directory is displayed below. The files highlighted in bold are used in one of the examples in the previous chapters. While all summary reports are created in the root directory and files from individual analyses are stored in the '`stats`' directory, all BAM files that may be required for additional downstream analyses (e.g., mapped and deduplicated BAM files) are stored in the '`out`' directory, organized by assigned family and sample.

```
├── nextflow/
│   ├── {DATE}_commands.txt
│   └── {CONFIG}
├── out/
│   └── {FAMILY}/
│       ├── 1-extracted/
│       │       └── {RG}_extractedReads-{FAMILY}.bam
│       └── best|fixed
│           ├── 2-aligned/
│           │   └── {RG}.{FAMILY}.{REF}.bam
│           ├── 3-deduped/
│           │   └── {RG}.{FAMILY}.{REF}_deduped.bam
│           ├── 4-bedfiltered/
│           │   └── {RG}.{FAMILY}.{REF}_deduped_bedfiltered.bam
│           ├── 5-deaminated/
│           │   ├── {RG}.{FAMILY}.{REF}_deduped_deaminated_1term.bam
│           │   └── {RG}.{FAMILY}.{REF}_deduped_deaminated_3term.bam
│           └── 6-mpielups/
│               ├── {RG}.{FAMILY}.{REF}_all_mpiled.tsv
│               ├── {RG}.{FAMILY}.{REF}_term1_mpiled.tsv
│               └── {RG}.{FAMILY}.{REF}_term3_mpiled.tsv
├── pipeline_versions.yml
├── R_final_report.tsv
├── filtered_report_{PSF}p_{PEB}b.tsv
├── final_report.tsv
└── stats/
    ├── splitcounts.tsv
    ├── {RG}_00_extracted.tsv
    ├── {RG}_01_mapped.tsv
    ├── {RG}_02_deduped.tsv
    ├── {RG}_03_bedfiltered.tsv
    ├── {RG}_04_deamination_positions/
    │      ├── {family}.{species}_substitutions.tsv
    │      ├── {family}.{species}_plot.jpg
    │      └── {family}.{species}_95CI-intervals.tsv
    ├── {RG}.kraken.report
    ├── {RG}.kraken.translate
    └── {RG}.krakenUniq.parsed_report.tsv
```

Extracted Reads
The 'ExtractedReads' are BAM files containing the binned sequences per family, based on the KrakenUniq classification. They are generated for each family assignment that passes the KrakenUniq filter thresholds (SI 4.1). The sequences in these files are unmapped.

Aligned Reads
The `'2-aligned'` directory contains all alignments generated by mapping the ExtractedReads of each family to all reference genomes under its "best" reference node, as identified from the KrakenUniq report. The alignment files include only mapped sequences and are not filtered for low-quality scores.

Deduped Reads
The `'3-deduped/'` directory contains the alignments from `'2-aligned/'`, with PCR duplicates removed. Sequences not passing the mapping-quality threshold are excluded. These BAM-files are the main input-files for additional downstream analyses (see SI 8.3.1).

Bedfiltered Reads
The `'4-bedfiltered/'` directory contains a single BAM file per family - the mapped and deduplicated sequences of the "final" best reference per family. Sequences overlapping low-complexity regions are removed based on the BED files provided with the quicksand database. By default, this directory is not generated for "fixed" references, as the fixed reference genomes lack the pre-generated BED files. The creation of a BED file and the bedfiltering-step can be enforced for 'fixed' references with the `--fixed_bedfiltering` flag.

Deaminated Reads
The `'5-deaminated/'` directory is created only for families with 'fixed' reference genomes. It contains BAM files with deaminated sequences from the deduplicated alignments (also bedfiltered if the `--fixed_bedfiltering` flag is set). For each alignment, two files are generated: one with sequences showing a terminal C-to-T or G-to-A substitution (1term), and another with sequences showing a C-to-T or G-to-A substitution within the first or last three base pairs (3term).

Mpileups
The `'6-mpileups/'` directory is created only for families with 'fixed' reference genomes. It contains the mapped and unique sequences as well as the deaminated sequences in mpileup-format for each fixed reference. The first three positions of each sequence are masked before the pileup by setting the mapping quality to 0.

References

Capo, Eric, Charline Giguet-Covex, Alexandra Rouillard, Kevin Nota, Peter D. Heintzman, Aurèle Vuillemin, Daniel Ariztegui, et al. 2021. "Lake Sedimentary DNA Research on Past Terrestrial and Aquatic Biodiversity: Overview and Recommendations". *Quaternary* 4 (1): 6. https://doi.org/10.3390/quat4010006.

Collin, Thomas C., Konstantina Drosou, Jeremiah Daniel O'Riordan, Tengiz Meshveliani, Ron Pinhasi, and Robin N. M. Feeney. 2020. "An Open-sourced Bioinformatic Pipeline for the Processing of Next-generation Sequencing Derived Nucleotide Reads: Identification and Authentication of Ancient Metagenomic DNA", unpublished data. https://doi.org/10.1101/2020.04.20.050369, last accessed October 10, 2025.

Consuegra, S., C. García De Leániz, A. Serdio, M. González Morales, L. G. Straus, D. Knox, and E. Verspoor. 2002. "Mitochondrial DNA Variation in Pleistocene and Modern Atlantic Salmon from the Iberian Glacial Refugium". *Molecular Ecology* 11 (10): 2037–48. https://doi.org/10.1046/j.1365-294x.2002.01592.x.

Courtin, Jérémy, Amedea Perfumo, Andrei A. Andreev, Thomas Opel, Kathleen R. Stoof-Leichsenring, Mary E. Edwards, Julian B. Murton, and Ulrike Herzschuh. 2022. "Pleistocene Glacial and Interglacial Ecosystems Inferred from Ancient DNA Analyses of Permafrost Sediments from Batagay Megaslump, East Siberia". *Environmental DNA* 4 (6): 1265–83. https://doi.org/10.1002/edn3.336.

Demarchi, Beatrice, Samantha Presslee, Igor Gutiérrez-Zugasti, Manuel González-Morales, Ana B. Marín-Arroyo, Lawrence G. Straus, and Roman Fischer. 2019. "Birds of Prey and Humans in Prehistoric Europe: A View from El Mirón Cave, Cantabria (Spain)". *Journal of Archaeological Science: Reports* 24 (April): 244–52. https://doi.org/10.1016/j.jasrep.2019.01.010.

Dimopoulos, Evangelos A., Alberto Carmagnini, Irina M. Velsko, Christina Warinner, Greger Larson, Laurent A. F. Frantz, and Evan K. Irving-Pease. 2022. "HAYSTAC: A Bayesian Framework for Robust and Rapid Species Identification in High-throughput Sequencing Data". Edited by Niranjan Nagarajan. *PLOS Computational Biology* 18 (9): e1010493. https://doi.org/10.1371/journal.pcbi.1010493.

Fellows Yates, James A., Christina Warinner, Aida Andrades Valtueña, Maxime Borry, Meriam Guellil, Alexander Herbig, Alina Hiß, et al. 2024. "Introduction to Ancient Metagenomics". Zenodo. https://doi.org/10.5281/ZENODO.13784555.

Gelabert, Pere, Susanna Sawyer, Anders Bergström, Ashot Margaryan, Thomas C. Collin, Tengiz Meshveliani, Anna Belfer-Cohen, et al. 2021. "Genome-scale Sequencing and Analysis of Human, Wolf, and Bison DNA from 25,000-year-old Sediment". *Current Biology* 31 (16): 3564–74.e9. https://doi.org/10.1016/j.cub.2021.06.023.

Günther, Torsten, Cristina Valdiosera, Helena Malmström, Irene Ureña, Ricardo Rodriguez-Varela, Óddny Osk Sverrisdóttir, Evangelia A. Daskalaki, et al. 2015. "Ancient Genomes Link Early Farmers from Atapuerca in Spain to Modern-day Basques". *Proceedings of the National Academy of Sciences* 112 (38): 11917–22. https://doi.org/10.1073/pnas.1509851112.

Hofreiter, Michael, Jim I. Mead, Paul Martin, and Hendrik N. Poinar. 2003. "Molecular Caving". *Current Biology* 13 (18): R693–95. https://doi.org/10.1016/j.cub.2003.08.039.

Hübler, Ron, Felix M. Key, Christina Warinner, Kirsten I. Bos, Johannes Krause, and Alexander Herbig. 2019. "HOPS: Automated Detection and Authentication of Pathogen DNA in Archaeological Remains". *Genome Biology* 20 (1). https://doi.org/10.1186/s13059-019-1903-0.

Kim, Daehwan, Li Song, Florian P. Breitwieser, and Steven L. Salzberg. 2016. "Centrifuge: Rapid and Sensitive Classification of Metagenomic Sequences". *Genome Research* 26 (12): 1721–29. https://doi.org/10.1101/gr.210641.116.

Kjær, Kurt H., Mikkel Winther Pedersen, Bianca De Sanctis, Binia De Cahsan, Thorfinn S. Korneliussen, Christian S. Michelsen, Karina K. Sand, et al. 2022. "A 2-million-year-old Ecosystem in Greenland Uncovered by Environmental DNA". *Nature* 612 (7939): 283–91. https://doi.org/10.1038/s41586-022-05453-y.

Korneliussen, Thorfinn Sand, Anders Albrechtsen, and Rasmus Nielsen. 2014. "ANGSD: Analysis of Next Generation Sequencing Data". *BMC Bioinformatics* 15 (1). https://doi.org/10.1186/s12859-014-0356-4.

Langmead, Ben, and Steven L Salzberg. 2012. "Fast Gapped-read Alignment with Bowtie 2". *Nature Methods* 9 (4): 357–59. https://doi.org/10.1038/nmeth.1923.

Liu, Sisi, Kathleen R. Stoof-Leichsenring, Lars Harms, Luise Schulte, Steffen Mischke, Stefan Kruse, Chengjun Zhang, and Ulrike Herzschuh. 2024. "Tibetan Terrestrial and Aquatic Ecosystems Collapsed with Cryosphere Loss Inferred from Sedimentary Ancient Metagenomics". *Science Advances* 10 (21). https://doi.org/10.1126/sciadv.adn8490.

Margaryan, Ashot, Henrik B. Hansen, Simon Rasmussen, Martin Sikora, Vyacheslav Moiseyev, Alexandr Khoklov, Andrey Epimakhov, et al. 2018. "Ancient Pathogen dna in Human Teeth and Petrous Bones". *Ecology and Evolution* 8 (6): 3534–42. https://doi.org/10.1002/ece3.3924.

Meyer, Matthias, Juan-Luis Arsuaga, Cesare de Filippo, Sarah Nagel, Ayinuer Aximu-Petri, Birgit Nickel, Ignacio Martínez, et al. 2016. "Nuclear DNA Sequences from the Middle Pleistocene Sima De Los Huesos Hominins". *Nature* 531 (7595): 504–7. https://doi.org/10.1038/nature17405.

Michelsen, Christian, Mikkel Winther Pedersen, Antonio Fernandez-Guerra, Lei Zhao, Troels C. Petersen, and Thorfinn Sand Korneliussen. 2022. "MetaDMG – A Fast and Accurate Ancient DNA Damage Toolkit for Metagenomic Data". unpublished data https://doi.org/10.1101/2022.12.06.519264, last accessed October 10, 2025.

Olalde, Iñigo, Morten E. Allentoft, Federico Sánchez-Quinto, Gabriel Santpere, Charleston W. K. Chiang, Michael DeGiorgio, Javier Prado-Martinez, et al. 2014. "Derived Immune and Ancestral Pigmentation Alleles in a 7,000-year-old Mesolithic European". *Nature* 507 (7491): 225–28. https://doi.org/10.1038/nature12960.

Olm, Matthew R., Alexander Crits-Christoph, Keith Bouma-Gregson, Brian A. Firek, Michael J. Morowitz, and Jillian F. Banfield. 2021. "Instrain Profiles Population Microdiversity from Metagenomic Data and Sensitively Detects Shared Microbial Strains". *Nature Biotechnology* 39 (6): 727–36. https://doi.org/10.1038/s41587-020-00797-0.

Oskolkov, Nikolay. 2025. "Refining Filtering Criteria for Accurate Taxonomic Classification of Ancient Metagenomic Data", unpublished data. https://doi.org/10.1101/2025.03.31.646431, last accessed October 10, 2025.

Ottoni, Claudio, Meriam Guellil, Andrew T. Ozga, Anne C. Stone, Oliver Kersten, Barbara Bramanti, Stéphanie Porcier, and Wim Van Neer. 2019. "Metagenomic Analysis of Dental Calculus in Ancient Egyptian Baboons". *Scientific Reports* 9 (1). https://doi.org/10.1038/s41598-019-56074-x.

O'Leary, Nuala A., Mathew W. Wright, J. Rodney Brister, Stacy Ciufo, Diana Haddad, Rich McVeigh, Bhanu Rajput, et al. 2015. "Reference Sequence (refseq) Database at NCBI: Current Status, Taxonomic Expansion, and Functional Annotation". *Nucleic Acids Research* 44 (D1): D733–45. https://doi.org/10.1093/nar/gkv1189.

Parducci, Laura, Kevin Nota, and Jamie Wood. 2018. "Reconstructing Past Vegetation Communities Using Ancient DNA from Lake Sediments". In *Paleogenomics*, 163–87. Springer International Publishing. https://doi.org/10.1007/13836_2018_38.

Pedersen, Mikkel W., Anthony Ruter, Charles Schweger, Harvey Friebe, Richard A. Staff, Kristian K. Kjeldsen, Marie L. Z. Mendoza, et al. 2016. "Postglacial Viability and Colonization in North America's Ice-free Corridor". *Nature* 537 (7618): 45–49. https://doi.org/10.1038/nature19085.

Pochon, Zoé, Nora Bergfeldt, Emrah Kırdök, Mário Vicente, Thijessen Naidoo, Tom van der Valk, N. Ezgi Altınışık, et al. 2023. "aMeta: An Accurate and Memory-efficient Ancient Metagenomic Profiling Workflow", *Genome Biol 24* (242). https://doi.org/10.1186/s13059-023-03083-9.

Prüfer, Kay, Cesare de Filippo, Steffi Grote, Fabrizio Mafessoni, Petra Korlević, Mateja Hajdinjak, Benjamin Vernot, et al. 2017. "A High-coverage Neandertal Genome from

Vindija Cave in Croatia”. *Science* 358 (6363): 655–58. https://doi.org/10.1126/science.aao1887.

Renaud, Gabriel, Udo Stenzel, and Janet Kelso. 2014. “Leehom: Adaptor Trimming and Merging for Illumina Sequencing Reads”. *Nucleic Acids Research* 42 (18): e141–41. https://doi.org/10.1093/nar/gku699.

Renaud, Gabriel, Kristian Hanghøj, Eske Willerslev, and Ludovic Orlando. 2016. “Gargammel: A Sequence Simulator for Ancient DNA”. Edited by Inanc Birol. *Bioinformatics* 33 (4): 577–79. https://doi.org/10.1093/bioinformatics/btw670.

Rognes, Torbjørn, Tomáš Flouri, Ben Nichols, Christopher Quince, and Frédéric Mahé. 2016. “VSEARCH: A Versatile Open Source Tool for Metagenomics”. *Peerj* 4 (October): e2584. https://doi.org/10.7717/peerj.2584.

Schulte, Luise, Nadine Bernhardt, Kathleen Stoof-Leichsenring, Heike H. Zimmermann, Luidmila A. Pestryakova, Laura S. Epp, and Ulrike Herzschuh. 2021. “Hybridization Capture of Larch (larix mill.) Chloroplast Genomes from Sedimentary Ancient DNA Reveals Past Changes of Siberian Forest”. *Molecular Ecology Resources* 21 (3): 801–15. https://doi.org/10.1111/1755-0998.13311.

Schönherr, Sebastian, Hansi Weissensteiner, Florian Kronenberg, and Lukas Forer. 2023. “Haplogrep 3 - an Interactive Haplogroup Classification and Analysis Platform”. *Nucleic Acids Research* 51 (W1): W263–68. https://doi.org/10.1093/nar/gkad284.

Simpson, Jared T., and Richard Durbin. 2010. “Efficient Construction of an Assembly String Graph Using the Fm-index”. Bioinformatics 26 (12): i367–73. https://doi.org/10.1093/bioinformatics/btq217.

Slon, Viviane, Charlotte Hopfe, Clemens L. Weiß, Fabrizio Mafessoni, Marco de la Rasilla, Carles Lalueza-Fox, Antonio Rosas, et al. 2017. “Neandertal and Denisovan DNA from Pleistocene Sediments”. *Science* 356 (6338): 605–8. https://doi.org/10.1126/science.aam9695.

Tejero, José-Miguel, Olivia Cheronet, Pere Gelabert, Brina Zagorc, Esteban Álvarez-Fernández, Pablo Arias, Aline Averbouh, et al. 2024. “Cervidae Antlers Exploited to Manufacture Prehistoric Tools and Hunting Implements as a Reliable Source of Ancient DNA”. *Heliyon* 10 (11): e31858. https://doi.org/10.1016/j.heliyon.2024.e31858.

Vasiliev, Sergei, Michael Shunkov, and Maxim Kozlikin. 2013. “Preliminary Results for the Balance of Megafauna from Pleistocene Layers of the East Gallery, Denisova Cave // Предварительные Итоги Изучения Остатков Мегафауны Из Плейстоценовых Отложений В Восточной Галерее Денисовой Пещеры”. *Problems of Archaeology, Ethnography, Anthropology of Siberia and Neighboring Territories - Novosibirsk* XIX: 32–38.

Vasiliev, Sergei, Michael Shunkov, and Maxim Kozlikin. 2017. "Megafaunal Remains from the Eastern Chamber of Denisova Cave and Problems of Reconstructing the Pleistocene Environments in the Northwestern Altai // Остатки Мегафауны Из Восточной Галереи Денисовой Пещеры И Вопросы Реконструкции Природной Среды Северо-западного Алтая В Плейстоцене". *Problems of Archaeology, Ethnography, Anthropology of Siberia and Neighboring Territories - Novosibirsk* XXIII: 60–64.

Vogel, Nicola Alexandra, Joshua Daniel Rubin, Mikkel Swartz, Juliette Vlieghe, Peter Wad Sackett, Anders Gorm Pedersen, Mikkel Winther Pedersen, and Gabriel Renaud. 2023. "Euka: Robust Tetrapodic and Arthropodic Taxa Detection from Modern and Ancient Environmental DNA Using Pangenomic Reference Graphs". *Methods in Ecology and Evolution* 14 (11): 2717–27. https://doi.org/10.1111/2041-210x.14214.

Wang, Yucheng, Mikkel Winther Pedersen, Inger Greve Alsos, Bianca De Sanctis, Fernando Racimo, Ana Prohaska, Eric Coissac, et al. 2021. "Late Quaternary Dynamics of Arctic Biota from Ancient Environmental Genomics". *Nature* 600 (7887): 86–92. https://doi.org/10.1038/s41586-021-04016-x.

Wang, Yucheng, Thorfinn Sand Korneliussen, Luke E. Holman, Andrea Manica, and Mikkel Winther Pedersen. 2022. "ngsLCA—a Toolkit for Fast and Flexible Lowest Common Ancestor Inference and Taxonomic Profiling of Metagenomic Data". *Methods in Ecology and Evolution* 13 (12): 2699–2708. https://doi.org/10.1111/2041-210x.14006.

Willerslev, Eske, Anders J. Hansen, Jonas Binladen, Tina B. Brand, M. Thomas P. Gilbert, Beth Shapiro, Michael Bunce, Carsten Wiuf, David A. Gilichinsky, and Alan Cooper. 2003. "Diverse Plant and Animal Genetic Records from Holocene and Pleistocene Sediments". *Science* 300 (5620): 791–95. https://doi.org/10.1126/science.1084114.

Xu, Jierui, Elena I. Zavala, and Priya Moorjani. 2025. "Sedimix: A Workflow for the Analysis of Hominin Nuclear DNA Sequences from Sediments", unpublished data. https://doi.org/10.1101/2025.02.28.640818, last accessed October 10, 2025.
